# Systems Analysis of the Liver Transcriptome in Adult Male Zebrafish Exposed to the Plasticizer (2-Ethylhexyl) Phthalate (DEHP)

**DOI:** 10.1038/s41598-018-20266-8

**Published:** 2018-02-01

**Authors:** Matthew Huff, Willian A. da Silveira, Oliana Carnevali, Ludivine Renaud, Gary Hardiman

**Affiliations:** 1 MUSC Bioinformatics, Center for Genomics Medicine, Medical University of South Carolina, Charleston, SC USA; 2 MS in Biomedical Sciences Program, Medical University of South Carolina, Charleston, SC USA; 3 Department of Pathology and Laboratory Medicine, Medical University of South Carolina, Charleston, SC USA; 4 Dipartimento di Scienze della Vita e dell’Ambiente, Università Politecnica delle Marche, 60131 Ancona, Italy; 5 Department of Medicine, Nephrology, Medical University of South Carolina, Charleston, SC USA; 6 Department of Medicine, University of California San Diego, La Jolla, CA USA; 7 Department of Public Health Sciences, Medical University of South Carolina, Charleston, SC USA; 8 Laboratory for Marine Systems Biology, Hollings Marine Laboratory, Charleston, SC USA

## Abstract

The organic compound diethylhexyl phthalate (DEHP) represents a high production volume chemical found in cosmetics, personal care products, laundry detergents, and household items. DEHP, along with other phthalates causes endocrine disruption in males. Exposure to endocrine disrupting chemicals has been linked to the development of several adverse health outcomes with apical end points including Non-Alcoholic Fatty Liver Disease (NAFLD). This study examined the adult male zebrafish (*Danio rerio*) transcriptome after exposure to environmental levels of DEHP and 17α-ethinylestradiol (EE2) using both DNA microarray and RNA-sequencing technologies. Our results show that exposure to DEHP is associated with differentially expressed (DE) transcripts associated with the disruption of metabolic processes in the liver, including perturbation of five biological pathways: ‘FOXA2 and FOXA3 transcription factor networks’, ‘Metabolic pathways’, ‘metabolism of amino acids and derivatives’, ‘metabolism of lipids and lipoproteins’, and ‘fatty acid, triacylglycerol, and ketone body metabolism’. DE transcripts unique to DEHP exposure, not observed with EE2 (i.e. non-estrogenic effects) exhibited a signature related to the regulation of transcription and translation, and ruffle assembly and organization. Collectively our results indicate that exposure to low DEHP levels modulates the expression of liver genes related to fatty acid metabolism and the development of NAFLD.

## Introduction

Endocrine Disrupting Compounds (EDCs) are ubiquitous chemical compounds used in numerous consumer products as plasticizers or flame retardants that have been shown to have unforeseen impacts on the ecosystem^1^ and human health^2^. As their chemical structures are similar to the structures of natural hormones^3^, EDCS are able to bind and activate many receptors, including nuclear hormone receptors^4,5^, and disrupt the endocrine system^6^. In light of these recent studies, EDCs are now considered chemicals of emerging concern^7^, and it is critical to fully understand the impact they might have on the health of the ecosystem and mankind at environmental levels.

A specific subset of EDCs, the xenoestrogens (XEs), are able to mimic 17β-estradiol, a natural female estrogen^8^. The effects of these XEs on specific cell types, including prostate, fibroblast, and neural cells, have recently been characterized^9,10^. However, their impact on the liver, the main site of detoxification and metabolism of xenobiotics^11^, has not been as well defined. Commonly used as a plasticizer of polyvinyl chloride (PVC), di(2-ethylhexyl) phthalate (DEHP), a XE gives PVC flexibility, strength and bendability, and is currently the only phthalate used in PVC medical devices^12,13^; the most highly DEHP exposed patients are neonates in the neonatal intensive care unit^14^. It is also present in office supplies and dust^15^ (notebooks, report covers and sheet protectors) and children’s toys^16^.

Two recent studies suggested that DEHP may cause lipid accumulation and nonalcoholic fatty liver disease (NAFLD) by promoting PPARα and sterol regulator element-binding protein 1c (SREBP-1c) expression^17,18^. Furthermore, exposure to DEHP has been found to disrupt the insulin signaling pathway in rats and the human L-02 cell line through activation of PPARγ^4^, reducing the ability of the liver to maintain glucose homeostasis, leading to insulin resistance.

In this study, we examined the effect of exposure to 5.8 nM of DEHP on the liver transcriptome, a concentration that is relevant to observed environmental levels^19^. In the United States, concentrations of DEHP in wastewater derived from Oakland, CA ranged from 2.53 to 6913.2 nM^20^. Studies in model organisms indicated that DEHP exposures ranging from 0.01 to 25.6 nM are sufficient to negatively affect animal growth and reproduction^21,22^.

To examine the effects of DEHP exposure on the adult male hepatic transcriptome, we exploited the zebrafish model (*Danio rerio*) and undertook a systems level analysis. We performed microarray and RNA sequencing analyses, and considered the data in the context of the recently described Adverse Outcomes Framework (AOF)^23^. This approach defines an adverse outcome as the end result of a causal series of events punctuated by “key events” (KE), the results of a molecular initiating event (MIE), a molecular interaction between a chemical stressor and a target biomolecule that alters gene expression. In parallel we assessed the effect of the E2 analogue 17α-ethinylestradiol (EE2) with the objective of deciphering the estrogenic and non-estrogenic effects of DEHP. This study is the first to use deep transcriptome profiling to explore the effects of exposure to DEHP on the zebrafish liver.

## Results

### Molecular changes in DEHP exposed livers revealed altered translational regulation

**Figure 1:**
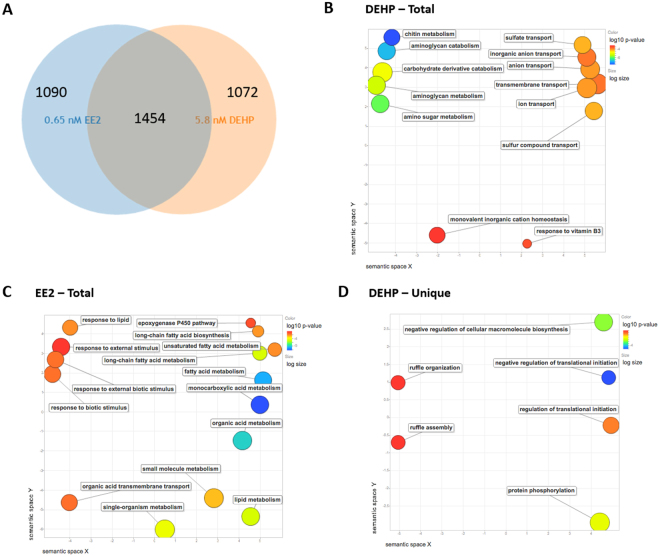
Functional Analyses. (**A**) Overlap of the top 3,000 ranked DE expressed liver transcripts from 5.8 nM DEHP and 0.65 nM EE2 exposed adult male zebrafish relative to control fish as determined by microarray analysis. (**B**–**D**) Gene Ontology Biological Process analyses: Scatterplots shows the cluster representatives (i.e. terms remaining after the redundancy reduction) in a two dimensional space derived by applying multidimensional scaling to a matrix of the GO terms’ semantic similarities. Bubble color indicates the p-value (legend in upper right-hand corner); size indicates the frequency of the GO term in the underlying GOA database (bubbles of more general terms are larger). GO BP analysis of DE genes in (**B**) DEHP and (**C**) EE2 exposed livers. (**D**) GO BP analysis of DE genes unique to DEHP (not DE in EE2 exposed).

To examine the effect of 21-day exposure to DEHP (5.8 nM) or EE2 (0.65 nM) on the liver of adult male zebrafish, we carried out a microarray experiment and analyzed the transcriptome of exposed and control fish. Unique array probes were ranked using an interest statistic described previously, which reflects the understanding that the gene with a greater absolute fold change is potentially more interesting^24–26^. Of the top 3,000 ranked DE transcripts, 1,454 mRNAs were shared amongst the EE2 and DEHP exposures. EE2 and DEHP exposures altered the expression of 1,090 and 1,072 unique mRNAs respectively (Fig. 1A). Next, we analyzed the top ranked 3,000 DE transcripts using the Gene Ontology (GO) enrichment analysis and visualization tool (GOrilla)^27^. DEHP exposure enriched a number of carbohydrate metabolism processes, including chitin metabolic and chitin catabolic processes (q = 4.46E-05 and 8.91E-05 respectively), amino sugar catabolic process (q = 1.16E-04) and ion transport (q = 3.63E-02) (Table 1, DEHP Total and Fig. 1B). Exposure to EE2 (Table 1, EE2 Total) was associated with enrichment in biological processes such as organic acid metabolic process (q = 9.31E-03), carboxylic acid metabolic processes (q = 1.14E-02), and oxoacid metabolic process (q = 1.16E-02), among other terms associated with lipid and fatty acid metabolism (Fig. 1C). We next performed enrichment analysis using DE gene signatures that were unique for DEHP and observed significant enrichment in terms related to regulation of translational initiation (q = 1.65E-01), negative regulation of translation (q = 2.48E-01), ruffle organization (q = 1.93E-01) and ruffle assembly (q = 2.14E-01) (Table 1, DEHP Unique and Fig. 1D). We also performed enrichment analysis using DE gene signatures unique to EE2, but found no significant enrichments (data not shown).

**Table 1:** GOrilla functional enrichment analysis.

GO Term	q-value
**DEHP-Total**	
chitin metabolic process	4.46E-05
chitin catabolic process	8.91E-05
amino sugar catabolic process	1.16E-04
glucosamine-containing compound metabolic process	1.39E-04
glucosamine-containing compound catabolic process	1.74E-04
aminoglycan catabolic process	2.31E-04
amino sugar metabolic process	8.38E-04
aminoglycan metabolic process	3.38E-03
carbohydrate derivative catabolic process	8.12E-03
sulfur compound transport	2.82E-02
sulfate transport	3.10E-02
anion transport	3.44E-02
ion transport	3.63E-02
inorganic anion transport	5.55E-02
transmembrane transport	5.71E-02
response to vitamin B3	1.28E-01
organonitrogen compound catabolic process	1.69E-01
monovalent inorganic cation homeostasis	2.07E-01
**EE2-Total**	
organic acid metabolic process	9.31E-03
monocarboxylic acid metabolic process	1.12E-02
carboxylic acid metabolic process	1.14E-02
oxoacid metabolic process	1.16E-02
fatty acid metabolic process	1.53E-02
long-chain fatty acid metabolic process	4.58E-02
lipid metabolic process	5.10E-02
single-organism metabolic process	5.51E-02
small molecule metabolic process	1.12E-01
unsaturated fatty acid metabolic process	1.25E-01
carboxylic acid transmembrane transport	1.30E-01
response to lipid	1.35E-01
long-chain fatty acid biosynthetic process	1.38E-01
organic acid transmembrane transport	1.39E-01
response to biotic stimulus	1.44E-01
response to external biotic stimulus	1.56E-01
epoxygenase P450 pathway	1.87E-01
monounsaturated fatty acid metabolic process	2.14E-01
response to external stimulus	2.23E-01
monounsaturated fatty acid biosynthetic process	2.26E-01
**DEHP-Unique**	
negative regulation of cellular biosynthetic process	1.35E-01
negative regulation of gene expression	1.44E-01
negative regulation of biosynthetic process	1.62E-01
regulation of translational initiation	1.65E-01
negative regulation of cellular macromolecule biosynthetic process	1.74E-01
negative regulation of translational intitiation	1.87E-01
protein phosphorylation	1.91E-01
ruffle organization	1.93E-01
ruffle assembly	2.14E-01
negative regulation of translation	2.48E-01
negative regulation of macromolecule biosynthetic process	2.62E-01
negative regulation of cellular amide metabolic process	2.71E-01

The top 3,000 ranked DE expressed liver transcripts as determined by microarray analysis from 5.8 nM DEHP and 0.65 nM EE2 exposed adult male zebrafish relative to control fish were used to search for enriched GO: Biological Process terms. The most significant terms for the DEHP and EE2 exposures, and those unique to DEHP (i.e. not present in in EE2 exposed) are presented.

### Functional enrichment analysis of human orthologs revealed altered transcriptional and translational pathways

**Figure 2:**
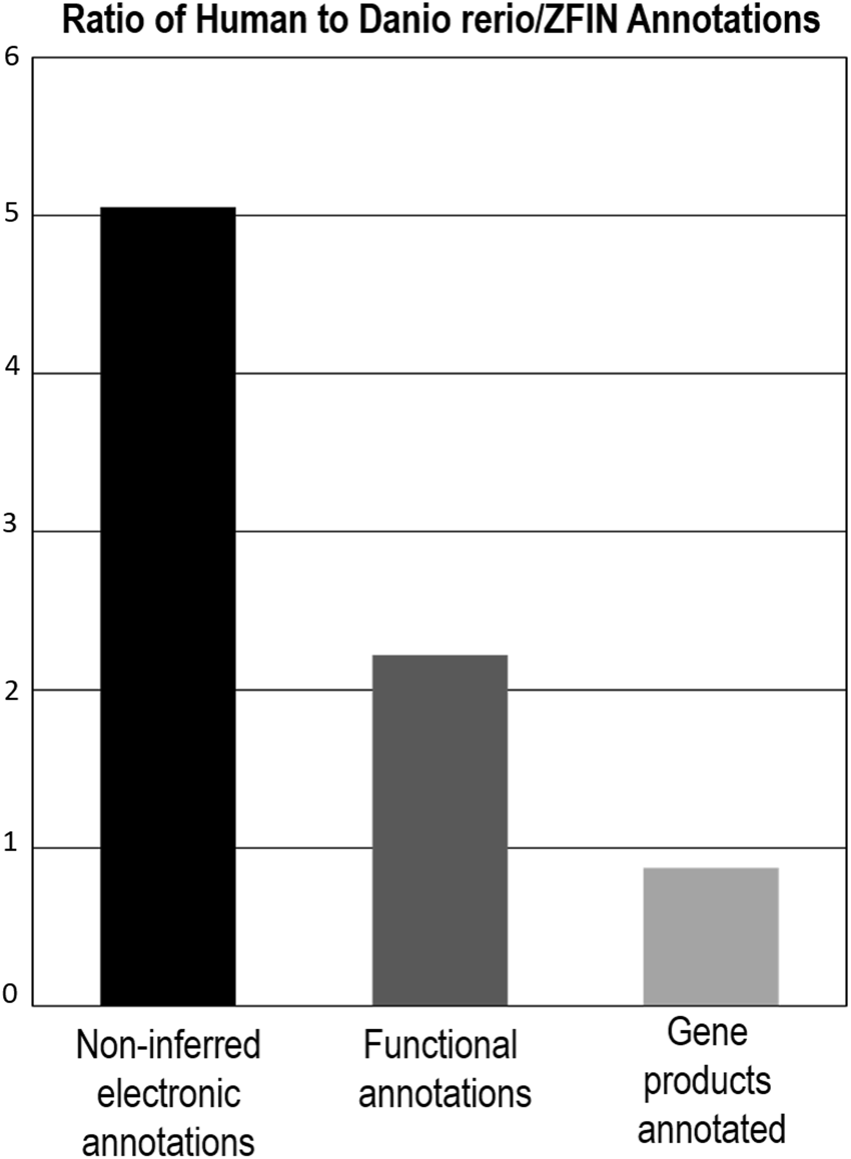
Functional annotations: (**A**) comparison of zebrafish and human annotations. Zebrafish has larger number of annotated gene products relative to human, 22,171 versus 19,392 (as of April 2017). In human, non-inferred electronic (black bar) and functional annotations (dark grey bar) are 5 times and 2 times better defined respectively than they are in zebrafish. In zebrafish, gene products annotated are slightly better defined than they are in human (light grey bar).

We mapped zebrafish genes of interest to their human orthologs using Ensembl^28,29^ genes to take advantage of the more extensive functional and non-inferred electronic annotations available based on the human genome, as illustrated in Fig. 2 ^6^. Using the assigned human orthologs, we performed enrichment analysis with the ‘Transcriptome, ontology, phenotype, proteome, and pharmacome annotations’ based gene list functional enrichment analysis (ToppFun) tool and observed that exposure to DEHP and EE2 both had a significant impact on metabolism (Table 2, Supplementary Tables S1–S4), including the organonitrogen compound biosynthetic process (DEHP Total, q = 3.87E-16) and oxoacid metabolic process (EE2 Total, q = 1.03E-14). Furthermore, exposures to EE2 and DEHP were both linked with significant enrichment relating to apoptotic processes (DEHP Total, programmed cell death, q = 2.76E-10 and EE2 Total, apoptotic process, q = 1.38E-11), while DE genes unique to DEHP were associated with enrichment in cell cycle-related processes (DEHP Total, q = 5.23E-11). DEHP exposure resulted in a unique enrichment signature related to RNA processing (DEHP Unique, q = 1.80E-04) and ribonucleoprotein complex biogenesis (DEHP Unique, q = 1.19E-03). In terms of co-expressed genes, we identified an overlap in the DE genes of interest to this study with genes that are up-regulated in the zebrafish crash & burn (crb) mutant for the bmyb gene (EE2 Total, q = 6.07E-21) and genes upregulated in hepatoblastoma (liver cancer cell) samples (DEHP Total, q = 2.26E-24). In DE genes expressed only after exposure to DEHP, we found commonality with genes up-regulated by activation of the mammalian target of rapamycin complex 1 (mTORC1) pathway (Table 2, DEHP Unique q = 8.83E-06, Supplementary Figure S1 and Supplementary Table S5).

**Table 2:** GO: Biological Process and Co-expression analysis.

GO Term	Bonferroni q-value
GO: Biological Process	
**DEHP - Total**	
organonitrogen compound biosynthetic process	3.87E-16
response to steroid hormone	3.91E-11
cell cycle process	5.23E-11
programmed cell death	2.76E-10
**EE2 - Total**	
response to endogenous stimulus	7.10E-16
oxoacid metabolic process	1.03E-14
peptide metabolic process	8.23E-12
apoptotic process	1.38E-11
**DEHP - Unique**	
cell cycle	2.00E-05
RNA processing	1.80E-04
positive regulation of transcription, DNA-templated	7.85E-04
ribonucleoprotein complex biogenesis	1.19E-03
**EE2 - Unique**	
negative regulation of transcription from RNA polymerase II promoter	2.56E-04
regulation of cell differentiation	1.94E-03
negative regulation of gene expression	1.22E-02
negative regulation of cellular biosynthetic process	1.09E-01
**Co-Expression**	
**DEHP - Total**	
Genes whose promoters are bound by MYC [GeneID = 4609], according to MYC	
Target Gene Database.	1.85E-24
Genes up-regulated in robust Cluster 2 (rC2) of hepatoblastoma samples	
compared to those in the robust Cluster 1 (rC1).	2.26E-24
Genes up-regulated through activation of mTORC1 complex.	3E-22
**EE2 - Total**	
Human Liver_Tzur09_1908genes	7.19E-22
Human orthologs of genes up-regulated in the crb (‘crash and burn’) zebrafish	
mutant that represents a loss-of-function mutation in BMYB [GeneID = 4605].	6.07E-21
**DEHP - Unique**	
Genes down-regulated in erythroid progenitor cells from fetal livers of E13.5	
embryos with KLF1 [GeneID = 10661] knockout compared to those from the wild type embryos.	4.59E-14
Genes up-regulated through activation of mTORC1 complex.	8.83E-06
**EE2 - Unique**	
Mouse Liver_White05_638genes	3.21E-06
Rat Liver_Perez-Carreon06_290genes	4.21E-04

The top 3,000 ranked DE expressed liver transcripts as determined by microarray analysis from 5.8 nM DEHP and 0.65 nM EE2 exposed adult male zebrafish relative to control fish were mapped to their human homologs using Ensembl homology. GO: Biological Process and co-expression terms and were enriched using ToppFun. The most significant terms for the DEHP and EE2 exposures, and those unique to DEHP (i.e. not present in in EE2 exposed) are presented.

Using ToppFun’s pathway analysis module to examine exposure to DEHP, we identified five pathways of interest (Table 3 and Supplementary Table S6): FOXA2 and FOXA3 transcription factor networks (Pathway ID = 137911), Metabolic pathways (132956), metabolism of amino acids and derivatives (1270158), metabolism of lipids and lipoproteins (1270001), and fatty acid, triacylglycerol, and ketone body metabolism (1270010). Our data suggests that DEHP influences expression of genes associated with metabolism, in particular the metabolism of lipids and fatty acids.

**Table 3:** DE genes from microarray analysis.

Zebra Entrez Gene ID	Probe ID	Gene Symbol	Gene Name	Human Entrez ID	Human Gene Symbol	Log2 ratio	FC
**FOXA2 and FOXA3** **transcription** **factor networks**							
449677	A_15_P117834	cpt1b	carnitine palmitoyltransferase 1B (muscle)	126129	*CPT1C*	−5.80	−55.72
30262	A_15_P115731	ins	preproinsulin	3630	*INS*	−3.61	−12.21
317638	A_15_P105778	igfbp1a	insulin-like growth factor binding protein 1a	3484	*IGFBP1*	−3.54	−11.63
30262	A_15_P110065	ins	preproinsulin	3630	*INS*	−3.26	−9.58
140815	A_15_P108996	cebpa	CCAAT/enhancer binding protein (C/EBP), alpha	1050	*CEBPA*	3.20	9.19
573723	A_15_P101211	acadvl	acyl-Coenzyme A dehydrogenase, very long chain	37	*ACADVL*	−2.51	−5.70
445118	A_15_P107918	g6pca	glucose-6-phosphatase a, catalytic	2538	*G6PC*	−2.46	−5.50
322493	A_15_P107681	slc2a2	solute carrier family 2 (facilitated glucose transporter), member 2	6514	*SLC2A2*	2.20	4.59
445118	A_15_P107270	g6pca	glucose-6-phosphatase a, catalytic”	2538	*G6PC*	−2.06	−4.17
325881	A_15_P112672	f2	coagulation factor II (thrombin)	2147	*F2*	0.10	1.07
**Fatty acid, triacylglycerol, and ketone body metabolism**							
436636	A_15_P111782	cd36	CD36 antigen	948	*CD36*	7.88	235.6
768196	A_15_P102657	me1	malic enzyme 1, NADP(+)-dependent, cytosolic	4199	*ME1*	6.01	64.45
386661	A_15_P112389	scd	stearoyl-CoA desaturase (delta-9-desaturase)	79966	*SCD5*	5.33	40.22
768196	A_15_P107967	me1	malic enzyme 1, NADP(+)-dependent, cytosolic	4199	*ME1*	5.23	37.53
393984	A_15_P119395	aacs	acetoacetyl-CoA synthetase	65985	*AACS*	5.00	32.00
317738	A_15_P108810	elovl6	ELOVL family member 6, elongation of long chain fatty acids (yeast)”	79071	*ELOVL6*	3.68	12.82
317738	A_15_P121489	elovl6	ELOVL family member 6, elongation of long chain fatty acids (yeast)	79071	*ELOVL6*	3.30	9.85
573723	A_15_P101211	acadvl	acyl-Coenzyme A dehydrogenase, very long chain	37	*ACADVL*	−2.51	−5.70
393622	A_15_P112797	acsl4a	acyl-CoA synthetase long-chain family member 4a	2182	*ACSL4*	2.38	5.21
327417	A_15_P109573	hsd17b12a	hydroxysteroid (17-beta) dehydrogenase 12a	51144	*HSD17B12*	2.20	4.5**9**
**Metabolic pathways**							
445818	A_15_P112409	cthl	cystathionase (cystathionine gamma-lyase), like”	1491	*CTH*	6.60	97.01
447879	A_15_P114717	zgc:103408	zgc:103408	27231	*NMRK2*	6.44	86.82
768196	A_15_P102657	me1	malic enzyme 1, NADP(+)-dependent, cytosolic	4199	*ME1*	6.01	64.45
768196	A_15_P107967	me1	malic enzyme 1, NADP(+)-dependent, cytosolic	4199	*ME1*	5.23	37.53
436799	A_15_P115180	atp6v1f	ATPase, H + transporting, V1 subunit F	9296	*ATP6V1F*	4.47	22.16
393799	A_15_P106842	pycr1	pyrroline-5-carboxylate reductase 1	5831	*PYCR1*	3.80	13.93
84039	A_15_P108167	bcmo1	beta-carotene 15,15’-monooxygenase 1	53630	*BCO1*	3.48	11.16
436919	A_15_P111364	ada	adenosine deaminase	100	*ADA*	3.39	10.48
406651	A_15_P117346	ddc	dopa decarboxylase	1644	*DDC*	3.31	9.92
406651	A_15_P111461	ddc	dopa decarboxylase	1644	*DDC*	3.31	9.92
**Metabolism of amino acids and derivatives**							
445818	A_15_P112409	cthl	cystathionase (cystathionine gamma-lyase), like”	1491	*CTH*	6.60	97.01
393799	A_15_P106842	pycr1	pyrroline-5-carboxylate reductase 1	5831	*PYCR1*	3.80	13.93
406651	A_15_P117346	ddc	dopa decarboxylase	1644	*DDC*	3.31	9.92
406651	A_15_P111461	ddc	dopa decarboxylase	1644	*DDC*	3.31	9.92
572649	A_15_P108171	zgc:112179	zgc:112179	8424	*BBOX 1*	−2.81	−7.01
114426	A_15_P109191	odc1	ornithine decarboxylase 1	4953	*ODC1*	2.14	4.41
30665	A_15_P120879	psmb9a	proteasome (prosome, macropain) subunit, beta type, 9a	5698	*PSMB9*	2.14	4.41
100000775	A_15_P111686	glula	glutamate-ammonia ligase (glutamine synthase) a	2752	*GLUL*	−2.13	−4.38
321892	A_15_P118448	ckmt1	creatine kinase, mitochondrial 1”	1159	*CKMT1B*	1.93	3.81
399488	A_15_P101243						
	zgc:55813	zgc:55813	6520	*SLC3A2*	−1.87	−3.66	
**Metabolism of lipids and lipoproteins**							
436636	A_15_P111782	cd36	CD36 antigen	948	*CD36*	7.88	235.57
768196	A_15_P102657	me1	malic enzyme 1, NADP(+)-dependent, cytosolic	4199	*ME1*	6.01	64.45
386661	A_15_P112389	scd	stearoyl-CoA desaturase (delta-9-desaturase)	79966	*SCD5*	5.33	40.22
768196	A_15_P107967	me1	malic enzyme 1, NADP(+)-dependent, cytosolic	4199	*ME1*	5.23	37.53
393984	A_15_P119395	aacs	acetoacetyl-CoA synthetase	65985	*AACS*	5.00	32.00
768298	A_15_P117841	faah2b	fatty acid amide hydrolase 2b	158584	*FAAH2*	3.83	14.22
317738	A_15_P108810	elovl6	ELOVL family member 6, elongation of long chain fatty acids (yeast)”	79071	*ELOVL6*	3.68	12.82
58128	A_15_P109314	fabp7a	fatty acid binding protein 7, brain, a	2173	*FABP7*	3.56	11.79
58128	A_15_P102880	fabp7a	fatty acid binding protein 7, brain, a	2173	*FABP7*	3.43	10.78
317738	A_15_P121489	elovl6	ELOVL family member 6, elongation of long chain fatty acids (yeast)	79071	*ELOVL6*	3.30	9.85

in adult male zebrafish exposed to 5.8 nM DEHP relative to controls associated with enriched biological pathways (Metabolic Pathways; Fatty acid, triacylglycerol, and ketone body metabolism; FOXA2 and FOXA3 transcription factor networks; Metabolism of amino acids and derivatives; Metabolism of Lipids and Lipoproteins). Genes with the greatest fold change difference in DEHP exposed relative to control are presented. An expanded list of DE genes is presented in Supplementary Table S6.

### RNA-Seq analysis identified changes in cellular response and translation

**Figure 3:**
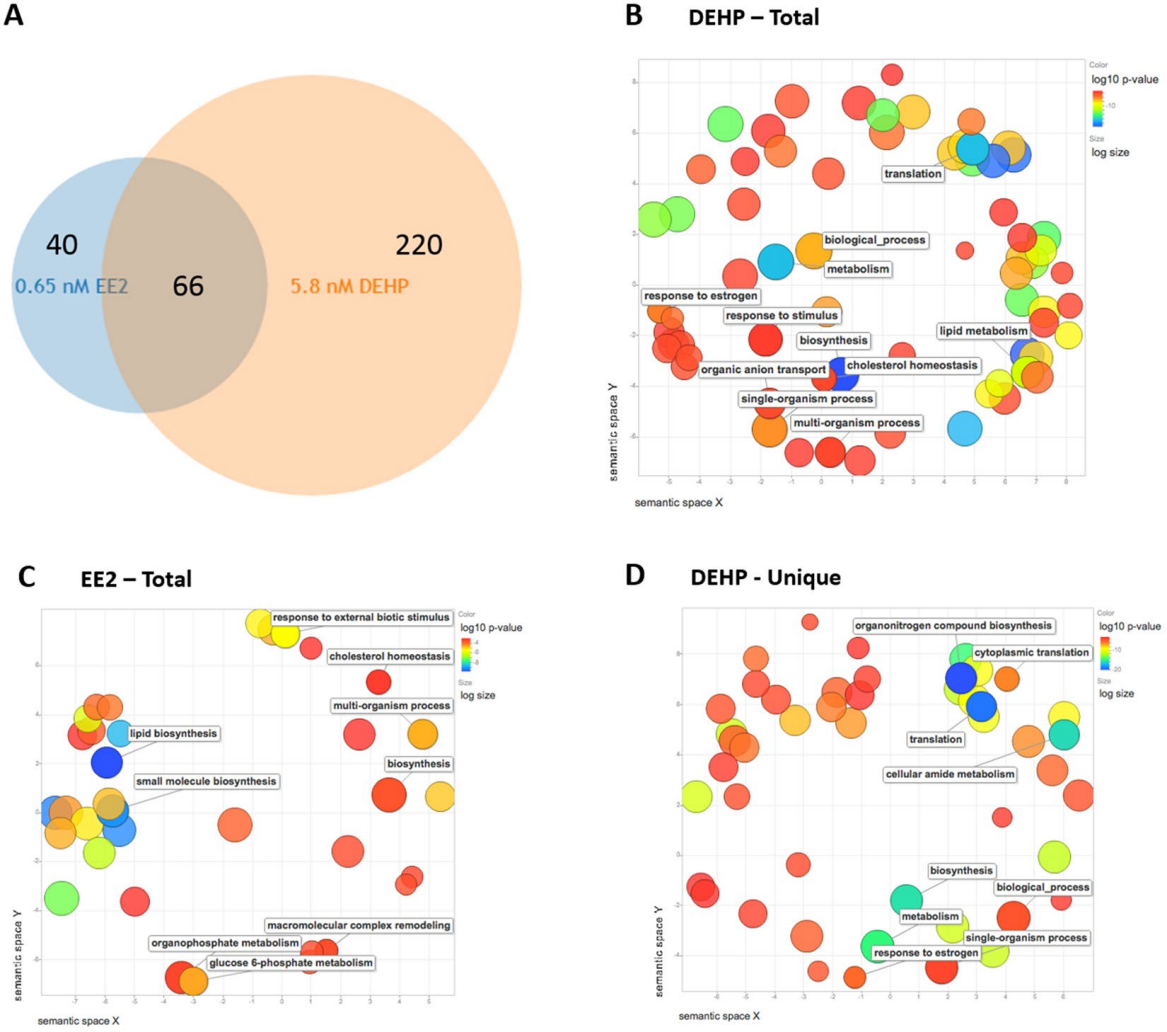
Functional Analyses RNA-Seq data. (**A**) Overlap of the significant DE expressed liver transcripts (FDR < 0.4) from 5.8 nM DEHP and 0.65 nM EE2 exposed adult male zebrafish relative to control fish as determined by DESeq. 2. (**B**–**D**) Gene Ontology Biological Process analyses: Scatterplots shows the cluster representatives (i.e. terms remaining after the redundancy reduction) in a two dimensional space derived by applying multidimensional scaling to a matrix of the GO terms’ semantic similarities. Bubble color indicates the p-value (legend in upper right-hand corner); size indicates the frequency of the GO term in the underlying GOA database (bubbles of more general terms are larger). GO BP analysis of DE genes in (**B**) DEHP and (**C**) EE2 exposed livers. (**D**) GO BP analysis of DE genes unique to DEHP (not DE in EE2 exposed).

High-throughput RNA-Seq was carried out to further explore transcriptomic changes in response to DEHP exposure using an advanced analytical pipeline we have recently described^30^. Based on DESeq2 analysis, genes with an adjusted *p*-value of less than or equal to 0.4 were selected^31–33^. We found that EE2 and DEHP altered the expression of 326 total genes; 66 genes were differentially expressed upon exposure to both DEHP and EE2, whereas exposures to EE2 and DEHP altered expression in 40 and 220 unique transcripts respectively (Fig. 3A).

Using these significant DE transcripts, we performed GOrilla analysis and found that exposures to both DEHP and EE2 were associated with enrichment of metabolic processes, particularly those relating to lipid metabolism (Table 4 and Fig. 3B,C), including lipid biosynthetic (DEHP Total, q = 7.01E-08 and EE2 Total, q = 8.58E-07) and metabolic processes (DEHP Total, q = 1.05E-08, EE2 Total, q = 2.58E-04) (all q-values are Bonferroni adjusted). Exposure to DEHP (DEHP Total, Table 4 and Fig. 3B) was associated with enrichments relating to translation (q = 6.09E-14), including cytoplasmic translation (q = 5.19E-04), and cellular responses to hypoxia (q = 1.24E-02) and response to estrogen (q = 2.94E-04). For exposure to EE2 (EE2 Total, Table 4 and Fig. 3C), we found unique enrichments related to the insulin signaling pathway (q = 1.03E-01) and the triglyceride metabolic pathway (q = 2.05E-02); the latter is supported with enrichments in triglyceride catabolism (q = 9.03E-02) and triglyceride-rich lipoprotein particle remodeling (q = 6.13E-02). Analysis of only the genes associated with exposure to DEHP (DEHP Unique, Table 4 and Fig. 3D) returned significant enrichment relating to translation (q = 3.10E-16), fatty acid biosynthesis (q = 1.26E-15), and the protein metabolic processes (q = 1.29E-02). No significant enrichments were detected with analysis of DE genes unique to EE2.

**Table 4:** RNA-Seq: GOrilla functional enrichment analysis.

GO Term	Bonferroni q-value
**DEHP Total**	
translation	6.09E-14
lipid metabolic process	1.05E-08
lipid biosynthetic process	7.01E-08
response to estrogen	2.94E-04
cytoplasmic translation	5.19E-04
response to hypoxia	1.24E-02
**EE2 - Total**	
lipid biosynthetic process	8.58E-07
lipid metabolic process	2.58E-04
triglyceride metabolic process	2.05E-02
triglyceride-rich lipoprotein particle remodeling	6.13E-02
triglyceride catabolic process	9.03E-02
regulation of insulin-like growth factor receptor signaling pathway	1.03E-01
**DEHP - Unique**	
translation	3.10E-16
fatty acid biosynthetic process	1.26E-15
protein metabolic process	1.29E-02

Significant DE expressed liver transcripts (FDR < 0.4) as determined by RNA-Seq from 5.8 nM DEHP and 0.65 nM EE2 exposed adult male zebrafish relative to control fish. The most significant terms for the DEHP and EE2 exposures, and those unique to DEHP (i.e. not present in in EE2 exposed) are presented.

### Functional enrichment analysis of RNA-seq data projected onto human orthologs identified dysregulation of translational and insulin-related pathways

We performed a functional enrichment analysis of the human orthologs using ToppFun and found that exposure to DEHP and EE2 is related to enrichment in metabolic processes (Table 5). In particular, EE2 enriched several lipid-related processes, including lipid biosynthesis (q = 3.87E-09) and fatty acid metabolism (q = 7.42E-08) (Table 5, EE2 Total; Supplementary Table S7). DEHP affected biological processes (Table 5, DEHP Total; Supplementary Table S8), such as co-translational protein localization to membranes (q = 1.01E-19), nuclear-transcribed mRNA catabolic process (q = 1.79E-16) and translational initiation (q = 2.84E-15). Analysis of genes unique to DEHP exposure supported these findings (Table 5, DEHP Unique): translation (q = 2.03E-16), processing of rRNA (q = 5.46E-14) and biogenesis of the ribonucleoprotein complex (q = 6.20E-11). Transcripts unique to EE2 were associated with enrichment in the triglyceride metabolic process (Table 5, EE2 Unique, q = 5.09E-01), response to dietary excess (q = 5.47E-01), and the neutral lipid metabolic process (q = 7.28E-01).

**Table 5:** RNA-Seq: GO: Biological Process and Co-expression analysis.

**GO Term**	**Bonferroni q-value**
**GO: Biological Process**	
**DEHP - Total**	
cotranslational protein targeting to membrane	1.01E-19
nuclear-transcribed mRNA catabolic process, nonsense-mediated decay	1.79E-16
translational initiation	2.84E-15
peptide biosynthetic process	6.43E-14
**EE2 - Total**	
small molecule biosynthetic process	2.27E-10
lipid biosynthetic process	3.87E-09
fatty acid metabolic process	7.42E-08
cholesterol metabolic process	9.28E-06
**DEHP - Unique**	
protein targeting to ER	2.86E-19
translation	2.03E-16
rRNA processing	5.46E-14
ribonucleoprotein complex biogenesis	6.20E-11
**EE2 - Unique**	
triglyceride metabolic process	5.09E-01
purine nucleoside monophosphate metabolic process	5.22E-01
response to dietary excess	5.47E-01
neutral lipid metabolic process	7.28E-01
**Co-Expression**	
**DEHP - Total**	
Genes up-regulated through activation of mTORC1 complex.	6.79E-14
Genes involved in cholesterol homeostasis.	6.58E-12
**EE2 - Total**	
Genes down-regulated in hepatocellular carcinoma (HCC) compared to normal liver samples.	3.12E-05
Genes encoding proteins involved in metabolism of fatty acids.	4.65E-04
**DEHP - Unique**	
Molecular timetable composed of 162 time-indicating genes	
(182 probes) in the peripheral (liver) clock.	2.49E-12
Genes up-regulated in liver from mice transgenic for SREBF1 or SREBF2 [GeneID = 6720, 6721] and down-regulated in mice lacking SCAP [GeneID = 22937].	4.78E-10

Significant DE expressed liver transcripts (FDR < 0.4) as determined by RNA-Seq from 5.8 nM DEHP and 0.65 nM EE2 exposed adult male zebrafish relative to control fish were mapped to their human homologs using Ensembl homology. GO: Biological Process and co-expression terms and were enriched using ToppFun. The most significant terms for the DEHP and EE2 exposures, and those unique to DEHP (i.e. not present in in EE2 exposed) are presented. Expanded lists are found in Supplementary Tables S7–S10.

In terms of Co-Expression (Supplementary Tables S9,10), in response to DEHP exposure (Table 5, DEHP total; Supplementary Table S10 and Figure S1), we observed an overlap with genes up-regulated with activation of the mTORC1 complex (q = 6.79E-14) and genes involved in cholesterol homeostasis (q = 6.58E-12).

**Figure 4:**
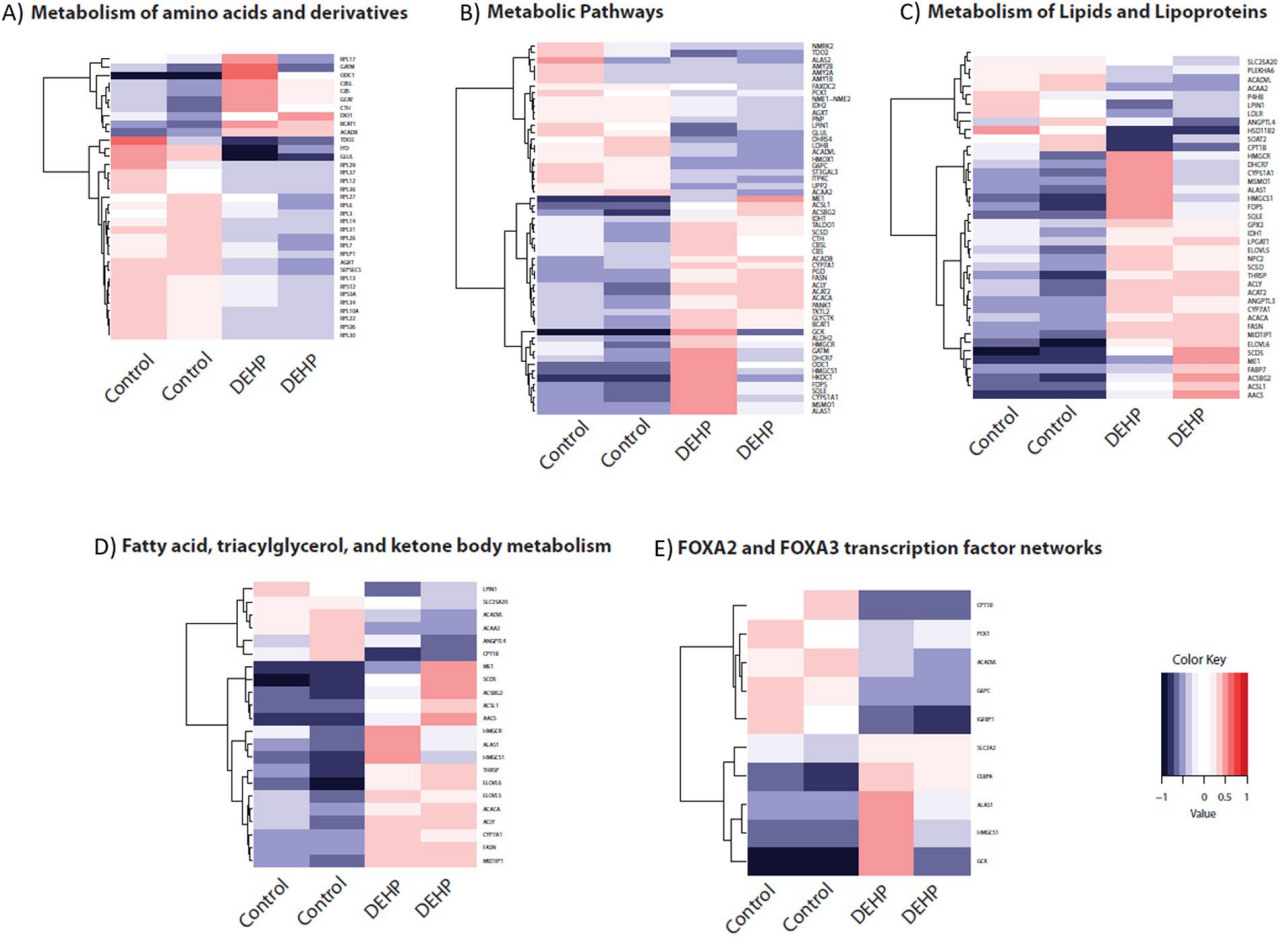
Heatmaps of significantly DE liver mRNAs as determined by DESeq. 2. (FDR < 0.4) in adult male zebrafish exposed to 5.8 nM DEHP relative to controls. DE genes associated with enriched biological pathways (**A**) Metabolism of amino acids and derivatives, (**B**) Metabolic Pathways, (**C**) Metabolism of Lipids and Lipoproteins, (**D**) Fatty acid, triacylglycerol, and ketone body metabolism and (**E**) FOXA2 and FOXA3 transcription factor networks are plotted. Red and blue boxes indicate relative over- and under-expression with respect to a reference which is calculated as the mid-point between the control and exposed groups.

As the mTORC1 complex is intricately associated with the Insulin-like growth factors (IGF-I and IGF-II) system that regulates metabolism we hypothesized that DEHP exposure could impact liver weight. In order to assess this functional effect we measured liver tissue weights from fish exposed to 100 nM DEHP, 100 nM EE2 or control ethanol exposure. This revealed reduction in the size of the liver in the DEHP exposed fish (Supplementary Figure S1). In response to EE2 exposure (Table 5; Supplementary Table S9), we observed co-expression of genes associated with fatty acid metabolism (q = 4.65E-04), as well as genes related to changes in expression observed in hepatocellular carcinoma (HCC) (q = 3.12E-05). Analysis of genes unique to exposure to DEHP (Table 5) identified an overlap with genes associated with the sterol regulatory element binding transcription factors (SREBFs) 1 and 2 and the SREBP cleavage-activating protein (SCAP) in the livers of transgenic mice (q = 4.78E-10). We found no significant overlap associated with the genes unique to exposure to EE2 (data not shown). Pathway analysis of genes DE expressed by exposure to DEHP revealed five pathways of interest (Supplementary Table S11): FOXA2 and FOXA3 transcription factor networks, metabolic pathways, metabolism of amino acids and derivatives, metabolism of lipids and lipoproteins, and fatty acid, triacylglycerol, and ketone body metabolism. We explored the expression patterns of the genes associated with these pathways in the control and the DEHP-exposed fish (Fig. 4) using heatmaps which show a clear signature associated with DEHP exposure.

## Discussion

Environmental chemicals can act through multiple toxicity pathways and induce adverse health outcomes. The relationship between a contaminant and a particular outcome in an individual is dependent on genetic background, target tissue, dose and other factors besides the mechanisms of action (MOA). Transitioning from current risk assessment practices to approaches based on big data collection and integration requires a paradigm shift in how this is executed. A significant challenge to risk assessment is accurately relating chemical impacts across species and stratifying effects or MOAs that are likely to be detrimental to human health. One recent strategy is the adverse outcome (AOP) pathways framework that organizes mechanistic and/or predictive relationships between initial chemical–biological interactions, pathways and networks, and adverse phenotypic outcomes^23^.

The goal of this study was to assess the effect of environmental levels of DEHP on the liver transcriptome in the adult male zebrafish using a systems level approach. Our analyses utilized a well described whole genome DNA microarray coupled to GOrilla analyses and demonstrated that DEHP deregulates carbohydrate metabolism, including chitin and aminoglycans, and protein synthesis. ToppFun analysis of the significant DE genes from the microarray experiments projected onto their human homologs suggested that DE genes shared by both DEHP/EE2 exposures mapped to pathways related to metabolism, the cell cycle, apoptosis and response to external stimuli. Additionally, DE transcripts unique to DEHP exposure exhibited a signature related to the regulation of transcription and translation. Co-expression analysis revealed overlap with gene expression patterns observed in apical endpoints such as liver cancer, and the up-regulation of genes in the mTORC1 pathway. An independent experiment exploited high throughput RNA-sequencing and this data supported these results, highlighting that DEHP exposure affected metabolic processes, particularly lipid metabolism. The DE mRNA signature unique to DEHP exposure was associated with GO terms related to gene expression, protein synthesis, lipid/fatty acid metabolism and RNA metabolism. Co-expression analysis again revealed similarity to genes linked with adverse liver disease outcomes. Finally, we compared microarray and RNA-Seq analyses, based on the projection of zebrafish genes onto their human orthologs and subsequent analysis using the ToppGene Suite for gene list enrichment analysis and the following pathway annotations; BioSystems: Pathway Interaction Database, BioSystems: REACTOME and BioSystems: KEGG. This comparison uncovered five pathways: ‘FOXA2 and FOXA3 transcription factor networks’, ‘Metabolic pathways’, ‘metabolism of amino acids and derivatives’, ‘metabolism of lipids and lipoproteins’, and ‘fatty acid, triacylglycerol, and ketone body metabolism’ shared between the microarray and sequencing experiments.

### The Benefits of Using Human Annotations in a Zebrafish Study

Presently the zebrafish genome is not as well characterized as the human genome and the level of functional annotation lags behind. However 70% of protein-coding human genes are related to genes found in the zebrafish and 84% of genes known to be associated with human disease have a zebrafish counterpart^34^. It is therefore worthwhile to consider the human orthologs of the zebrafish genes in systems level analyses. As shown in Fig. 2^6^ in terms of the ratio of human to zebrafish annotations, there are 5 times more non-inferred electronic and 2.2 times more functional annotations in the human genome relative to the zebrafish (based on the Gene Ontology Consortium data April 2017). Utilizing GO terms for human orthologs provides a deeper systems level analysis than that obtained with zebrafish genes. We achieved a greater number of enriched terms in mRNA signatures unique to EE2 using human orthologs: enrichment analysis of the zebrafish genes in GOrilla uncovered no significant results, whereas analysis of the corresponding human orthologs with ToppFun identified significant enriched terms. A limitation to this approach is the absence of specific genes, for example vitellogenins (VTGs) which do not have orthologs in humans^21^. However for a comparative analysis between zebrafish and humans, however, these limitations are outweighed by the benefits of improved annotations and the comprehensive systems analysis afforded.

### Exposure to both DEHP and EE2 alter metabolic processes in the liver

Our data suggest that exposure to both EE2 and DEHP leads to the differential expression of genes involved in metabolic pathways representing molecular initiating events (MIEs). Since the liver is the primary site of metabolism within the body, the enrichment of metabolic pathways in both the DEHP and EE2 exposures was not unexpected. Among these common enrichment terms were changes to organic acid metabolism, in particular carboxylic acid metabolism^35^.

Co-expression results from ToppFun analysis identified overlaps between the DE mRNAs and the human orthologs of genes upregulated in the zebrafish crb (“crash and burn”) mutant, this mutation induces a loss-of-function of the gene *bmyb*, a transcription factor^36^. Loss of bmyb function can cause genome instability, and adult crb zebrafish heterozygotes have an increased cancer susceptibility^36^. Our results show that exposure to both DEHP and EE2 can mimic the effects of *bmyb* inactivation, suggesting a possible role for both molecules in carcinogenesis.

### Unique effects of DEHP exposure indicate links with non-alcoholic fatty liver disease (NAFLD)

The list of significant DE mRNAs overlaps with genes up-regulated in the liver of transgenic mice overexpressing sterol regulatory element binding transcription factors 1 and 2 (*SREBF1* and *SREBF2)*, and with low levels of SREBP cleavage-activating protein (*SCAP)* chaperone^37^. SREBF facilitates the development of NAFLD by increasing the synthesis of fatty acids within hepatocytes^38^. SCAP is required for the activation of SREBPs, and knockout of *SCAP* in mice has been shown to reduce the rate of fatty acid synthesis in the liver^39,40^. Another xenoestrogen bisphenol A (BPA) was shown recently to produce hepatosteatosis in zebrafish and human hepatocytes by up-regulating the endocannabinoid system. Hepatosteatosis, was associated with an increase in the liver levels of the obesogenic endocannabinoids 2-arachidonoylglycerol and anandamide and a concomitant decrease in palmitoylethanolamide^41^. Furthermore, we recently demonstrated that chronic BPA exposure impacts the hepatic epigenome in adult male zebrafish with altered gene expression signatures associated with non-alcoholic fatty liver disease (NAFLD) and cell cycle^42^. Similar results have been obtained in our laboratory with di-isononyl phthalate (DiNP) which upregulates orexigenic signals and causes hepatosteatosis together with deregulation of the peripheral endocannabinoid system (ECS) and lipid metabolism^43^.

### Non-estrogenic effects of DEHP relate to changes in translation and membrane ruffling

When we analyzed DEHP’s global effects on the liver transcriptome, in parallel we assessed the effects of EE2. This allowed us to identify the estrogenic effects of DEHP, as well as separate non-estrogenic effects by considering DE mRNAs unique to DEHP exposure and therefore not regulated by EE2. Analysis of these unique mRNAs identified the non-estrogenic effects of DEHP exposure. GO analysis of zebrafish terms indicated that DEHP uniquely induced changes in translational initiation, a signature not observed with EE2 exposure. This is supported by data from both the microarray and RNA-Seq experiments.

Co-expression analysis of genes unique to DEHP exposure supported the connection between translational changes and DEHP exposure. We observed that genes DE in response to DEHP exposure overlapped with genes up-regulated in relation to the mTORC1 pathway; mTORC1 regulates protein synthesis by accepting growth factors to activate translation, consequently making it a major effector of cell growth and proliferation^44,45^. Dysregulation of mTORC1 is associated with the development of an increasing number of pathologies, including cancer^46^, obesity^47,48^, type 2 diabetes^49,50^, and NAFLD^51,52^. Along with Akt and S6K1, mTORC1 is a key component of the insulin signaling pathway, and has been identified as an enhancer of SREBP1c’s role as master transcription factor in lipid synthesis^53^; mTORC1 regulates SREBP by controlling the nuclear entry of a phosphatidic acid phosphatase called lipin 1, which mediates the effects of mTORC1 on SREBP target gene, SREBP promoter activity, and nuclear SREBP protein abundance^52^. Intriguingly, hyperactivity of mTORC1 in liver cells has been shown to protect against steatosis in mice^51^; deletion of *Tsc1* in mouse liver cells, which codes hamartin (TSC1) of the tuberous sclerosis complex (TSC) and controls the activity of mTORC1^54^, specifically protected cells from high-fat diet induced, Akt-mediated steatosis through restriction of S6K1 independent of Akt suppression^51^.

We examined the most significantly deregulated mRNAs that are part of the mTORC1 pathway. *PPP1R15A*, *LDLR*, *PNP*, *IFRD1*, *NUPR1*, *SSR1* and *PNO1* were all downregulated in response to DEHP exposure. Protein Phosphatase 1 Regulatory Subunit (*PPP1R15A*) mRNA levels are increased following stressful growth arrest conditions^55^. The low density lipoprotein receptor (*LDLR*) gene encodes a cell surface protein involved in receptor-mediated endocytosis of specific ligands^56^. Purine Nucleoside Phosphorylase (*PNP*) encodes an enzyme which reversibly catalyzes the phosphorolysis of purine nucleosides^57^. Interferon Related Developmental Regulator 1 (*IFRD1*) is an immediate early gene that encodes a protein related to interferon-gamma, it can function as a transcriptional co-activator/repressor and control the growth and differentiation of specific cell types during embryonic development and tissue regeneration^58^. Nuclear Protein 1 Transcriptional Regulator (*NUPR1*) encodes a chromatin-binding protein that converts stress signals into a program of gene expression that empowers cells with resistance to the stress induced by a change in their microenvironment^59^. The signal sequence receptor 1 (*SSR1*) is a glycosylated endoplasmic reticulum (ER) membrane receptor associated with protein translocation across the ER membrane^60^. *ELOVL6*, *HMGCR*, *DHCR7*, *CYP51A1*, *HMGCS1*, *ME1*, *SQLE*, *SC5D*, *CTH*, *BCAT1*, *ACLY*, *ELOVL5*, *ACACA*, *HSPA5*, *HSPD1*, *IDH1* and *ELOVL6* were all upregulated in response to DEHP exposure. Fatty Acid Elongase 6 (*ELOVL6*) uses malonyl-CoA as a 2-carbon donor in the first and rate-limiting step of fatty acid elongation^61^. 3-Hydroxy-3-Methylglutaryl-CoA Reductase (*HMGCR*) is the rate-limiting enzyme for cholesterol synthesis. 7-Dehydrocholesterol Reductase (*DHCR7*) encodes an enzyme that removes the C(7-8) double bond in the B ring of sterols and catalyzes the conversion of 7-dehydrocholesterol to cholesterol. Cytochrome P450 Family 51 Subfamily A Member 1 (*CYP51A1*) encodes a member of the cytochrome P450 superfamily and participates in the synthesis of cholesterol by catalyzing the removal of the 14alpha-methyl group from lanosterol. 3-Hydroxy-3-Methylglutaryl-CoA Synthase 1 (*HMGCS1*) is an enzyme that condenses acetyl-CoA with acetoacetyl-CoA to form HMG-CoA, which is the substrate for HMG-CoA reductase. Malic Enzyme 1 (*ME1*) encodes a cytosolic, NADP-dependent enzyme that generates NADPH for fatty acid biosynthesis. The activity of this enzyme, the reversible oxidative decarboxylation of malate, links the glycolytic and citric acid cycles. Among its related pathways are Metabolism and Regulation of lipid metabolism by Peroxisome proliferator-activated receptor alpha (PPARalpha). Squalene Epoxidase (*SQLE*) catalyzes the first oxygenation step in sterol biosynthesis. Sterol-C5-Desaturase (*SC5D*) encodes an enzyme of cholesterol biosynthesis. Branched Chain Amino Acid Transaminase 1 (*BCAT1*) encodes the cytosolic form of the enzyme branched-chain amino acid transaminase. ATP Citrate Lyase (*ACLY*) is the primary enzyme responsible for the synthesis of cytosolic acetyl-CoA in many tissues. ELOVL Fatty Acid Elongase (*ELOVL5*) encodes a multi-pass membrane protein that is localized in the endoplasmic reticulum and is involved in the elongation of long-chain polyunsaturated fatty acids. Acetyl-CoA Carboxylase Alpha (*ACACA*) is regulated by the phosphorylation/dephosphorylation of targeted serine residues and by allosteric transformation by citrate or palmitoyl-CoA. Heat Shock Protein Family A (*Hsp70*) Member (HSPA5) is localized in the lumen of the endoplasmic reticulum (ER), and is involved in the folding and assembly of proteins in the ER^62^. As this protein interacts with many ER proteins, it may play a key role in monitoring protein transport through the cell. Heat Shock Protein Family D (*Hsp60*) Member 1 (HSPD1) encodes a member of the chaperonin family.

We observed a reduction in the size of the liver in adult zebrafish in response to a seven day 100 nM DEHP exposure (Supplementary Figure S1). The mTOR signaling pathway senses and integrates a variety of environmental cues to regulate organismal growth and homeostasis^45^. DEHP exposure in the nematode *Caenorhabditis elegans* was found to be acutely toxic, with decreases in body length and egg number per worm observed after 24 h of DEHP exposure^63^. DEHP has also been shown to decrease serum estradiol levels and aromatase expression, prolong estrous cycles, and cause anovulation in animal and culture models and directly. It inhibits antral follicle growth via a mechanism that partially includes reduction in levels of estradiol production and decreased expression of cell cycle regulators^64^.

Other significant GO terms unique to DEHP exposure included ruffle assembly and organization - the formation of actin rich membrane protrusions typically used for cell motility^65^. In the context of the liver, ruffles form in response to insulin signaling. Mice with the fatty liver dystrophy (*fld*) mutation lose the ability to properly form membrane ruffles, the result of impairments in insulin-mediated cytoskeleton dynamics^66^. One of the hallmarks of this mutation is the formation of a fatty liver, similar to what is observed in NAFLD^67^. Furthermore, in muscle cells, defective actin remodeling is associated with the development of insulin resistance^68^; defined as the inability of cells to be regulated by insulin signaling, insulin resistance is associated with adverse outcomes such as type 2 diabetes, cardiovascular disease, and chronic liver disease^69–71^. Recently, exposure to DEHP has been implicated with disruption of the insulin signaling pathway of both rats and the human cell line L-02 by activating PPARG, reducing the liver’s ability to maintain glucose homeostasis and contributing to insulin resistance^4^. The significant enrichment of an insulin-mediated pathway – ruffle organization – supports the role of DEHP as a disrupter of insulin function, including the potential to inhibit insulin signaling in liver cells. Analysis of the signatures unique to DEHP exposure has identified changes related to translation and membrane ruffling; combined with our co-expression analysis, these perturbations support an association between DEHP exposure and NAFLD.

### Comparison of microarray and RNA-Seq platforms

Both the microarray and RNA-Seq analyses of the transcriptome identified perturbations in gene expression. While RNA-Seq is a newer more reproducible technology with less background noise, and a greater dynamic range to measure gene expression, DNA microarrays are still commonly utilized due to their cost and ease^30^. In the context of our results, we were interested in comparing microarray and sequencing analyses from the two different experiments where zebrafish were exposed to 5.8 nM DEHP. In general, we found that the results of our sequencing analysis supported those of our microarray analysis; many of the same GO and co-expression terms were enriched in both analyses, including genes up-regulated with the loss of *bmyb* and changes in translation initiation in response to exposure to DEHP.

In addition, the sequencing analysis uncovered GO terms that strengthen the connection between DEHP exposure and the development of adverse outcomes in the liver. For example, analysis of the microarray data derived signatures unique to DEHP exposure identified enrichments in membrane ruffling, a process regulated by insulin in liver cells^72^. Analysis of the RNA-Seq data determined that exposure to DEHP enriched the same set of genes associated with changes in regulation of *SREBF1* and *SREBF2*. These transcription factors are significant in the development of liver pathologies and insulin resistance, and support previous studies associating exposure to DEHP with dysregulation of the insulin pathway^4^. Taken together, the combined genomic platforms identified molecular initiating and key events in the liver transcriptome that are conducive to the development of insulin resistance.

### Bone morphogenic protein 2 (BMP2) and the epigenetic effects of DEHP exposure

Analysis of DEHP’s unique mRNA signature obtained from the microarray experiment identified an overlap with genes that were down-regulated in the uterus upon knockdown of bone morphogenic protein 2 (BMP2) (Supplementary Table S3). In the uterus, BMP signaling is necessary for embryonic development, and BMP2 in particular is critical for embryonic implantation by inducing decidualization^73^, a rapid remodeling of uterine endometrial stroma into epithelial decidual cells that is critical for the progress of implantation^74^. Knockout of *BMP2* in mice renders mothers infertile due to lack of signaling to the stromal cells to begin decidualization^73^. Outside of the uterus, exposure to DEHP has been associated with decreased expression of BMPs, including BMP2, in dam mesenchymal stem cells^75^. DEHP mediates its adverse effects by interfering with signaling mechanisms involved in oocyte growth (VTG), maturation via the bone morphogenetic protein-15 (BMP15), luteinizing hormone receptor (LHR), membrane progesterone receptors (mPRs) and ovulation (cyclooxygenase (COX)−2 (ptgs2)), thereby deeply impairing ovarian functions^76^; female zebrafish exposed to environmentally relevant doses of DEHP exhibited a significant decrease in fecundity with diminished rates of ovulation and embryo production.

DEHP causes epigenetic effects impacting gene expression in the developing testis^77^. Alterations in DNA methylation patterns caused by maternal exposure have been indicated to play a key role in DEHP-mediated testicular toxicity. Maternal exposure to DEHP at 500 mg/kg/d induces testicular dysgenesis syndrome in fetuses and embryos^78^. DEHP exposure has also been shown to significantly inhibit gap-junctional intercellular communication, likely providing the initial stimulus that enables cell transformation, and facilitates development of these cells into tumors^79^. *In utero* DEHP exposure delays maturation of fetal Leydig cells, with reduced mineralocorticoid receptor (MR; NR3C2) expression in the adult Leydig cell^80^. Activation of MR induces androgen synthesis^81^, whereas its inhibition blocks testosterone synthesis in the Leydig cell^82^. Interestingly, it has been shown that DNA methylation and histone modification play a role in the regulation of BMP2^83,84^. Taken together, these studies suggest that DEHP could regulate the expression of BMP2 via epigenetic modification.

## Conclusion

The focus of this study was to examine the global effects of exposure to environmental levels of the plasticizer DEHP in the adult male zebrafish and uncover perturbations that represent MIEs that can potentially lead to adverse outcomes. Our results indicate that exposure to DEHP and EE2 leads to the expression of liver genes related to the mTORC1 complex, fatty acid metabolism and the development of non-alcoholic fatty liver disease. Analysis of only the DE genes associated with exposure to DEHP reveals potentially non-estrogenic perturbations in eukaryotic translation and insulin signaling after exposure. This data supports previous studies that implicate DEHP as increasing the risk of developing NAFLD.

## Materials and Methods

### Ethinylestradiol and (2-ethylhexyl) phthalate exposure studies in zebrafish

For both the microarray and RNA-Seq experiments adult male zebrafish (*Danio rerio*) were housed in aquaria that were individually heated using a 100 W aquarium heater to maintain a temperature of 26–29 °C, and the light–dark cycle was 14:10 h. The water pH ranged from 7.0 to 7.6 throughout the duration of the experiment. Aeration and filtration were provided using sponge filters. Fish were fed two times a day with commercial flaked fish food (Tetra, Germany). Fish were acclimated for one week prior to commencing the experiments. Three tanks (80 l/tank) with 40 animals each were prepared for the different experimental groups, one containing water with 5.8 nM of (2-ethylhexyl) phthalate (DEHP), one tank containing water with 0.65 nM of ethinylestradiol 17-α (EE2), and one tank containing water and ethanol as negative exposure control. The levels of DEHP are considered “environmentally relevant^19^,” and previous studies have determined that exposing model organisms to these concentrations is associated with altered effects on the transcriptome^21,76,85,86^. All chemicals were dissolved in ethanol and stock working solutions were prepared, from which the working experimental concentrations were prepared. The nominal exposures utilized a continuous flow-through system. Following a 21 day exposure the fish were sacrificed and sampled for liver tissue. Tissue samples were immediately frozen in liquid nitrogen and stored at −70 °C. For the liver mass experiments, animals were exposed to 100 nM of DEHP or EE2 for seven days, and the animals were sampled for liver tissue. All procedures involving zebrafish were performed in accordance with The University of California San Diego, Institutional Animal Care and Use Committee (IACUC) guidelines. All the experiments described above were approved by the IACUC and were performed in accordance with institutional guidelines and regulations.

### RNA Extraction

Isolation of total RNA from zebrafish liver samples was performed using TRIzol reagent (Invitrogen) and the extracted RNA were further purified using the RNeasy Mini kit (Qiagen, Valencia, CA). All RNA was subjected to on-column digestion of DNA during RNA purification from cells, to ensure highly pure RNA free from DNA contamination. The concentrations were determined by absorbance readings (OD) at 260 nm using an ND-1000 (Nanodrop, Wilmington, DE). RNA was further assessed for integrity with the 6000 Nano LabChip assay from Agilent, (Santa Clara, CA). Only RNA samples with a RIN score of >7.0 were used for genomic analyses. There were 18 samples in total (six EE2 exposed, six DEHP exposed, six control fish), selected for RNA extraction.

### Microarray Analysis

Using the Low RNA Input Fluorescent Linear Amplification Kit (Agilent), 100 ng of total RNA were converted into fluorescently labeled Cy3 cRNA. Unincorporated nucleotides of fluorescent targets were removed using RNeasy (Qiagen). Absorbance (OD) at 260 nm was used to quantify cRNA concentrations, and absorbance at 550 nm was used to measure Cy3 dye incorporation. Microarray hybridization was only carried out with cRNA that had an incorporation efficiency of 9 pmol/µg or greater.

We utilized the Agilent *D*. *Rerio* Oligo Microarray 4 × 44 K G2519F (015064), array design A-MEXP-1396 (Santa Clara, CA). cRNA target hybridization to the zebrafish microarray was carried out in accordance with single color Agilent Hybridization protocols, and have been described previously^25^. Array data were collected using an Agilent Microarray Scanner and Feature Extraction Software (v10.5), and deposited in the ArrayExpress Database, accession number E-TABM-547. Though Agilent’s Feature Extraction Software (v10.5) provides high quality expression reports for microarrays, the data still needed to be normalized to remove background technical noise and other subtle biases caused by hybridization. For this experiment, statistical analysis of the microarray experiment involved two steps: normalization and sorting of genes according to interest. All samples were normalized simultaneously using the multiple-loess technique^87^. Log ratios for each probe (technical replicates) were calculated separately, then averaged over the biological replicate microarrays. The data was sorted using the interest statistic, which reflects the understanding that the gene with a greater absolute fold change is potentially more interesting, (which we have described in greater detail previously)^24–26^. The design of the interest statistic was based on ideas borrowed from the software package Focus^88^.

### RNA sequencing (RNA-Seq)

To prepare RNA-Seq libraries using the TruSeq RNA Sample Prep Kit (Illumina, San Diego, CA), 100–200 ng of total RNA was used following the protocol described by the manufacturer. High throughput sequencing (HTS) was performed using an Illumina GAIIX with each sample sequenced to a minimum depth of ~2 million reads. A single end 50 cycle sequencing strategy was employed. Data were subjected to Illumina quality control (QC) procedures (>80% of the data yielded a Phred score of 30). RNA-Seq data has been submitted to the NCBI Gene Expression Omnibus, accession number GSE100367. Secondary analysis was carried out on an OnRamp Bioinformatics Genomics Research Platform (OnRamp Bioinformatics, San Diego, CA)^30^. OnRamp’s advanced Genomics Analysis Engine utilized an automated RNA-seq workflow to process the data^89,90^, including (1) data validation and quality control, (2) read alignment to the zebrafish genome (GRCz10) using TopHat2^91^, which revealed >73% mapping, (3) generation of gene-level count data with HTSeq.^92^, and (4) differential expression analysis with DEseq. 2^93^. Transcript count data from DESeq. 2 analysis of the samples were sorted according to their adjusted p-value (or q-value), which is the smallest false discovery rate (FDR) at which a transcript is called significant. FDR is the expected fraction of false positive tests among significant tests and was calculated using the Benjamini-Hochberg multiple testing adjustment procedure^30,31,93,94^.

### Gene Ontology and Pathway Analyses

The Gene Ontology Enrichment Analysis and Visualization Tool (GOrilla) was used to search enriched GO terms associated with DEHP and EE2 exposures^27,95^. Data was further analyzed with the GO summarization tool REViGO^96^ which combines redundant terms into a single, representative term based on a simple clustering algorithm relying on semantic similarity measures. We exploited Ensembl homology to append a human gene ID to a given zebrafish gene ID, in order to permit systems analysis using the ‘Transcriptome, ontology, phenotype, proteome, and pharmacome annotations based gene list functional enrichment analysis’ (Toppfun) tool and the richer Gene Ontology content available for human compared to zebrafish^6^. ToppFun sources content from multiple databases, including KEGG, WikiPathways, and REACTOME^97–99^. To ensure that only the most relevant terms were selected, we applied Bonferroni FDR correction.

## Electronic supplementary material

Supplementary Information

Supplementary Dataset 2

Supplementary Dataset 3

Supplementary Dataset 4
