# Supplementary material for: Systems Analysis of the Liver Transcriptome in Adult Male Zebrafish Exposed to the Plasticizer (2-Ethylhexyl) Phthalate (DEHP)
Source: Sci Rep. 2018 Feb 1;8:2118. doi: 10.1038/s41598-018-20266-8 (PMC5794889; doi:10.1038/s41598-018-20266-8)
Supplement: Supplementary file 1 — Supplementary Information [file 41598_2018_20266_MOESM1_ESM.pdf]

SUPPLEMENTARY INFORMATION

SYSTEMS ANALYSIS OF THE LIVER TRANSCRIPTOME IN ADULT MALE ZEBRAFISH EXPOSED TO THE PLASTICIZER (2-ETHYLHEXYL) PHTHALATE (DEHP).

Matthew Huff[1,2], Willian da Silveira[1,3], Oliana Carnevali[4], Ludivine Renaud[5], Gary Hardiman[1,5,6]

[1]MUSC Bioinformatics, Center for Genomics Medicine, Medical University of South Carolina, Charleston, SC 29415

[2]MS in Biomedical Sciences Program, Medical University of South Carolina

[3]Department of Pathology and Laboratory Medicine, Medical University of South Carolina

[4]Dipartimento Scienze della Vita e dell'Ambiente, Universita Politecnica delle Marche, 60131 Ancona, Italy.

[5]Departments of Medicine and Public Health Sciences, Medical University of South Carolina

[6]Laboratory for Marine Systems Biology, Hollings Marine Laboratory, Charleston, SC 29412

Correspondence should be addressed to Gary Hardiman; hardiman@musc.edu

TABLE OF CONTENTS

Figure S1

Table S1

Table S2

Table S3

Table S4

Table S5

Table S6

Table S7

Table S8

Table S9

Table S10

Table S11

Figure S1

A)

Co-Expression Term - Genes up-regulated through activation of mTORC1 complex.		
**Platform**	**Exposure**	**Bonferroni q-value**
Array: Top 3000 ranked DE genes in the livers of zebrafish exposed to DEHP	DEHP	3.00E-22
RNA-seq: DE genes differentially expressed in the livers of zebrafish exposed to DEHP (FDR <0.4)	DEHP	6.79E-14
RNA-seq: DE genes differentially expressed in the livers of zebrafish exposed to EE2 (FDR <0.4)	EE2	6.34E-07

B)

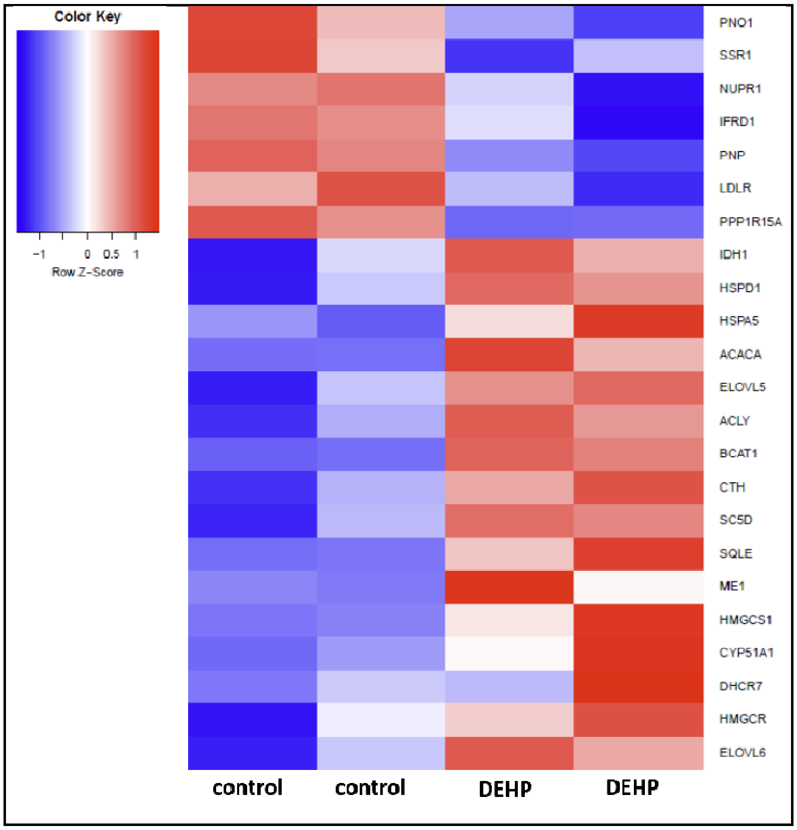

C)

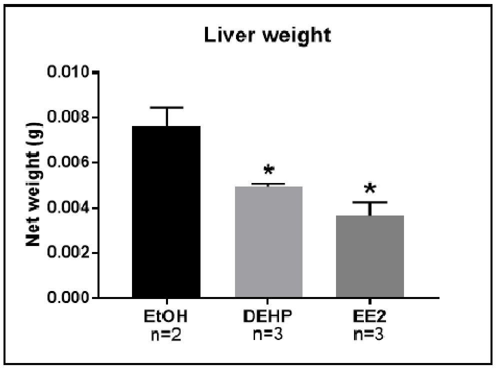

Figure S1 (LEGEND)

A. Genes up-regulated through activation of mTORC1 complex. HALLMARK_MTORC1_SIGNALING signature was provided by the MSigDB database (http://software.broadinstitute.org/gsea/msigdb/cards/HALLMARK_MTORC1_SIGNALING.html). The genomics platform used, DEHP or EE2 exposure and Bonferroni q-value for the the resulting co-expression signatures are provided.
B. Heatmaps of significantly expressed DE liver mRNAs (FDR <0.4) in adult male zebrafish exposed to 5.8 nM DEHP relative to controls, that belong to the HALLMARK_ MTORC1_ SIGNALING signature as determined by DESeq2 and ToppFun analysis.
C. Liver mass was determined from ETOH control AND 100 nM EE2 and DEHP exposed zebrafish, following a one week exposure. Statistical analyses were performed using GraphPad Prism, i.e. 1-way ANOVA analysis (p=0.0013) with Dunnett's post-test (DEHP versus EtOH, p=0.005, EE2 versus EtOH, p=0.0008).

Supplemental Table S1. Human Entrez gene IDs were mapped to zebrafish via Ensembl protein homology. Gene enrichment analysis was performed utilizing the ToppGene suite's functional enrichment tool, ToppFun. The Top 50 GO: Biological Process enrichments for genes differentially expressed in the livers of zebrafish exposed to EE2 are displayed.

GO Term	q-value Bonferroni
oxidation-reduction process	5.11E-18
single-organism biosynthetic process	3.51E-17
carboxylic acid metabolic process	6.75E-16
response to endogenous stimulus	7.10E-16
response to hormone	3.27E-15
organic acid metabolic process	4.42E-15
organonitrogen compound biosynthetic process	4.73E-15
oxoacid metabolic process	1.03E-14
cellular amide metabolic process	7.80E-14
purine ribonucleoside metabolic process	8.09E-14
purine nucleoside metabolic process	1.56E-13
response to oxygen-containing compound	6.34E-13
ribonucleoside metabolic process	1.80E-12
nucleoside metabolic process	6.26E-12
response to organic cyclic compound	8.14E-12
peptide metabolic process	8.23E-12
apoptotic process	1.38E-11
glycosyl compound metabolic process	1.95E-11
nucleobase-containing small molecule metabolic process	2.95E-11

purine-containing compound metabolic process	3.35E-11
purine ribonucleoside monophosphate metabolic process	3.45E-11
nucleoside monophosphate metabolic process	3.68E-11
purine nucleoside monophosphate metabolic process	4.35E-11
ribonucleoside monophosphate metabolic process	5.63E-11
programmed cell death	8.02E-11
nucleoside phosphate metabolic process	9.43E-11
nucleotide metabolic process	2.51E-10
response to organonitrogen compound	3.34E-10
regulation of cell death	4.14E-10
central nervous system development	8.14E-10
organophosphate metabolic process	8.36E-10
amide biosynthetic process	1.05E-09
regulation of apoptotic process	1.14E-09
regulation of programmed cell death	1.59E-09
purine nucleotide metabolic process	3.05E-09
cellular amino acid metabolic process	4.57E-09
purine ribonucleotide metabolic process	5.18E-09
response to abiotic stimulus	5.63E-09
regulation of cell proliferation	6.97E-09
ribonucleotide metabolic process	8.60E-09
nucleoside triphosphate metabolic process	9.34E-09
lipid metabolic process	1.05E-08
purine nucleoside triphosphate metabolic process	1.27E-08

response to nitrogen compound	1.49E-08
gland development	1.55E-08
peptide biosynthetic process	1.65E-08
translation	1.77E-08
head development	2.59E-08
ribose phosphate metabolic process	3.01E-08
positive regulation of transcription from RNA polymerase II promoter	4.97E-08

Supplemental Table S2. Human Entrez gene IDs were mapped to zebrafish via Ensembl protein homology. Gene enrichment analysis was performed utilizing the ToppGene suite's functional enrichment tool, ToppFun. The Top 50 GO: Biological Process enrichments for genes differentially expressed in the livers of zebrafish exposed to DEHP are displayed.

GO Term	q-value Bonferroni
single-organism biosynthetic process	2.14E-25
carboxylic acid metabolic process	4.16E-20
organic acid metabolic process	8.89E-20
oxoacid metabolic process	2.17E-19
organonitrogen compound biosynthetic process	3.87E-16
response to hormone	3.13E-15
response to abiotic stimulus	3.53E-15
oxidation-reduction process	1.22E-14
response to endogenous stimulus	7.50E-14
cell cycle	7.54E-13
positive regulation of biosynthetic process	4.42E-12
mitotic cell cycle	9.63E-12
cellular amide metabolic process	1.06E-11
positive regulation of cellular biosynthetic process	1.50E-11
response to steroid hormone	3.91E-11
cell cycle process	5.23E-11
positive regulation of nitrogen compound metabolic process	5.40E-11
positive regulation of nucleobase-containing compound metabolic process	1.03E-10
cellular amino acid metabolic process	1.23E-10
mitotic cell cycle process	1.62E-10

apoptotic process	2.35E-10
programmed cell death	2.76E-10
small molecule biosynthetic process	5.25E-10
DNA metabolic process	5.73E-10
regulation of cell death	6.87E-10
positive regulation of macromolecule biosynthetic process	7.25E-10
organophosphate metabolic process	7.35E-10
peptide metabolic process	7.56E-10
positive regulation of gene expression	1.84E-09
regulation of programmed cell death	2.96E-09
nucleoside phosphate metabolic process	2.97E-09
lipid metabolic process	4.44E-09
response to oxygen-containing compound	4.47E-09
cellular catabolic process	4.48E-09
regulation of cell cycle	5.58E-09
amide biosynthetic process	5.92E-09
regulation of apoptotic process	6.29E-09
nucleobase-containing small molecule metabolic process	6.78E-09
regulation of cell proliferation	6.79E-09
generation of precursor metabolites and energy	7.25E-09
nucleotide metabolic process	7.78E-09
ncRNA metabolic process	8.30E-09
response to oxidative stress	9.80E-09
monocarboxylic acid metabolic process	1.01E-08

positive regulation of RNA biosynthetic process	1.11E-08
negative regulation of cell death	1.80E-08
positive regulation of RNA metabolic process	1.93E-08
nucleoside metabolic process	3.13E-08
organic hydroxy compound metabolic process	3.15E-08
regulation of catabolic process	3.29E-08

Supplemental Table S3. Human Entrez gene IDs were mapped to zebrafish via Ensembl protein homology. Co-expression analysis was performed using ToppFun and the top 3000 ranked DE genes in the livers of zebrafish exposed to EE2. The Top 50 co-expressed gene sets are displayed.

Co-Expression	q-value Bonferroni
Mouse StemCell_Lindmark04_2514genes	1.55E-56
Mouse StemCell_Schimmer06_1554genes	3.87E-40
Human Leukemia_Yukinawa06_2000genes	6.05E-36
Mouse Viral_Mendoza-Villanueva08_1830genes	5.31E-33
Human Kidney_Sallustio10_2134genes_DiscriminatedARPCsFromRPTEC/MSC	1.83E-32
Human Kidney_Sallustio10_2134genes_CompleteListAnalysis	4.38E-32
Genes up-regulated in CD34+ [GeneID=947] cells isolated from bone marrow of CML (chronic myelogenous leukemia) patients, compared to those from normal donors.	3.92E-31
Mouse StemCell_Lindmark04_950genes	1.00E-30
Mouse InnerEar_Sajan08_2230genes	5.56E-29
Genes constituting the BRCA1-PCC network of transcripts whose expression positively correlated (Pearson correlation coefficient, PCC >= 0.4) with that of BRCA1 [GeneID=672] across a compendium of normal tissues.	1.87E-28
Mouse StemCell_Chambers07_1667genes	3.47E-28
Human Immune_Kong10_2491genes_ImmPort_CD40LandAnti-IgMvsControl	1.94E-27
Human Breast_Parker09_1918genes_IntrinsicGenes_Compilation	1.42E-26
Human Ovarian_Li09_2322genes_cisplatinresistance	3.47E-26
Human EmbryonicStemCell_Thomas08_1088genes	2.31E-25
Rat Hypothalamic_Mansuy10_1931genes	6.20E-25
Human StemCell_Nuytten08_2072genes	1.69E-23
Genes down-regulated in brain from patients with Alzheimer's disease.	2.00E-23

Human Colon_Grade07_1950genes	7.00E-22
Human Liver_Tzur09_1908genes	7.19E-22
Mouse Liver_White05_638genes	1.14E-21
Mouse Lymphoma_Wu08_1813genes	1.57E-21
Human Breast_van'tVeer02_2460genes_Ergenes	3.54E-21
Human orthologs of genes up-regulated in the crb ('crash and burn') zebrafish mutant that represents a loss-of-function mutation in BMYB [GeneID=4605].	6.07E-21
Human Breast_Creighton08_2154genes	7.08E-21
Human Immune_Kong10_1790genes_ImmPort_Anti-IgMvsControl	1.43E-20
Genes whose expression significantly and positively correlated with oligodendrocyte density in layer VI of BA9 brain region in patients with bipolar disorder.	7.47E-20
Human Breast_Mutarelli08_1488genes	1.33E-18
Genes down-regulated in uterus upon knockout of BMP2 [GeneID=650].	1.38E-18
Genes down-regulated in erythroid progenitor cells from fetal livers of E13.5 embryos with KLF1 [GeneID=10661] knockout compared to those from the wild type embryos.	1.98E-18
Genes up-regulated in ME-A cells (breast cancer) undergoing apoptosis in response to doxorubicin [PubChem=31703].	3.00E-18
Genes up-regulated in differentiating C2C12 cells (myoblasts) upon expression of PPARGC1A [GeneID=10891] off an adenoviral vector.	4.91E-18
Human StemCell_Nuytten08_1622genes	5.03E-18
Human Brain_Lu06_1128genes	5.79E-18
Mouse Lung_Rangasamy09_2003genes	9.51E-18
Human Breast_Teschendorff07_813genes_ERneg	1.22E-17
Mouse StemCell_Lawlor06_1431genes	1.86E-17
Genes down-regulated in intestinal crypt cells upon deletion of CTNNB1 [GeneID=1499].	2.15E-17
Mouse Lymphoma_Wu08_1148genes	3.19E-17

Genes down-regulated in ALL (acute lymphoblastic leukemia) blasts after 1 week of treatment with glucocorticoids.	4.52E-17
Human Lymphoma_Lam08_1502genes	8.77E-17
Mouse StemCell_Ramos06_1482genes	1.21E-16
Mouse Breast_Stein09_1780genes	1.24E-16
Genes up-regulated in robust Cluster 2 (rC2) of hepatoblastoma samples compared to those in the robust Cluster 1 (rC1).	2.40E-16
Human Viral_Cairo08_982genes	2.72E-16
The 'adult tissue stem' module: genes coordinately up-regulated in a compendium of adult tissue stem cells.	6.25E-16
Genes up-regulated in NIH3T3 cells (fibroblasts) transformed by expression of contitutively active (Q63L) form of RHOA [GeneID=387] off plasmid vector.	6.57E-16
Mitochondrial genes	1.01E-15
Genes commonly down-regulated in human alveolar rhabdomyosarcoma (ARMS) and its mouse model overexpressing PAX3-FOXO1 [GeneID=5077;2308] fusion.	1.64E-15
Human BoneMarrow_Zhan07_2181genes	1.72E-15

Supplemental Table S4. Human Entrez gene IDs were mapped to zebrafish via Ensembl protein homology. Co-expression analysis was performed using ToppFun and the top 3000 ranked DE genes in the livers of zebrafish exposed to DEHP. The Top 50 co-expressed gene sets are displayed.

Co-Expression	q-value Bonferroni
Mouse StemCell_Lindmark04_2514genes	1.93E-53
Mouse StemCell_Schimmer06_1554genes	7.49E-47
Rat Hypothalamic_Mansuy10_1931genes	1.30E-43
Genes up-regulated in CD34+ [GeneID=947] cells isolated from bone marrow of CML (chronic myelogenous leukemia) patients, compared to those from normal donors.	1.20E-40
Genes constituting the BRCA1-PCC network of transcripts whose expression positively correlated (Pearson correlation coefficient, PCC >= 0.4) with that of BRCA1 [GeneID=672] across a compendium of normal tissues.	1.15E-39
Human Leukemia_Yukinawa06_2000genes	2.32E-37
Genes down-regulated in uterus upon knockout of BMP2 [GeneID=650].	5.68E-34
Mouse Viral_Mendoza-Villanueva08_1830genes	3.60E-33
Genes up-regulated in NIH3T3 cells (fibroblasts) transformed by expression of constitutively active (Q63L) form of RHOA [GeneID=387] off plasmid vector.	1.20E-32
Genes constituting the CHEK2-PCC network of transcripts whose expression positively correlates (Pearson correlation coefficient, PCC >= 0.4) with that of CHEK2 [GeneID=11200].	4.83E-32
Human orthologs of genes up-regulated in the crb ('crash and burn') zebrafish mutant that represents a loss-of-function mutation in BMYB [GeneID=4605].	7.27E-31
Human Breast_Mutarelli08_1488genes	1.09E-30
Mouse Lung_Rangasamy09_2003genes	3.22E-30

Human Ovarian_Li09_2322genes_cisplatinresistance	5.90E-30
Mouse StemCell_Lindmark04_950genes	7.79E-30
Human Immune_Kong10_2491genes_ImmPort_CD40LandAnti-IgMvsControl	7.87E-30
Human Kidney_Sallustio10_2134genes_DiscriminatedARPCsFromRPTEC/MSC	1.44E-28
Genes up-regulated in TC71 and EWS502 cells (Ewing's sarcoma) by EWSR1-FLI1 [GeneID=2130;2314] as inferred from RNAi knockdown of this fusion protein.	3.35E-28
Genes down-regulated in erythroid progenitor cells from fetal livers of E13.5 embryos with KLF1 [GeneID=10661] knockout compared to those from the wild type embryos.	3.67E-28
Human Kidney_Sallustio10_2134genes_CompleteListAnalysis	7.94E-28
Mouse Lymphoma_Wu08_1167genes	1.31E-27
Genes down-regulated in brain from patients with Alzheimer's disease.	2.71E-27
Human EmbryonicStemCell_Thomas08_1088genes	1.78E-26
Genes down-regulated in intestinal crypt cells upon deletion of CTNNB1 [GeneID=1499].	2.12E-26
Human Breast_Parker09_1918genes_IntrinsicGenes_Compilation	1.17E-24
Genes whose promoters are bound by MYC [GeneID=4609], according to MYC Target Gene Database.	1.85E-24
Human Viral_Cairo08_982genes	1.90E-24
Genes up-regulated in robust Cluster 2 (rC2) of hepatoblastoma samples compared to those in the robust Cluster 1 (rC1).	2.26E-24
Human StemCell_Hassan09_1544genes	4.48E-24
Genes down-regulated in nasopharyngeal carcinoma (NPC) compared to the normal tissue.	6.60E-24
Human Liver_Tzur09_1908genes	1.08E-23

Mouse StemCell_Ramos06_1482genes	1.26E-23
Human Breast_Creighton08_2154genes	1.15E-22
The 'core ESC-like gene module': genes coordinately up-regulated in a compendium of mouse embryonic stem cells (ESC) which are shared with the human ESC-like module.	1.19E-22
Genes up-regulated through activation of mTORC1 complex.	3.00E-22
Human Breast_Teschendorff07_813genes_ERneg	3.52E-22
Genes down-regulated in APL (acute promyeolocytic leukemia) blasts expressing PML-RARA fusion [GeneID=5371;5914] compared to normal promyeloblasts.	9.37E-22
Genes up-regulated in poorly differentiated thyroid carcinoma (PDTC) compared to normal thyroid tissue.	1.09E-21
Human Colon_Grade07_1950genes	2.62E-21
Genes whose promoters contain E-box motifs and whose expression changed in MYCN-3 cells (neuroblastoma) upon induction of MYCN [GeneID=4613].	6.07E-21
Mouse InnerEar_Sajan08_2230genes	8.68E-21
Genes up-regulated in differentiating C2C12 cells (myoblasts) upon expression of PPARGC1A [GeneID=10891] off an adenoviral vector.	1.43E-20
Genes up-regulated in ME-A cells (breast cancer) undergoing apoptosis in response to doxorubicin [PubChem=31703].	2.51E-20
Human Breast_van'tVeer02_2460genes_Ergenes	3.49E-20
Human BoneMarrow_Zhan07_2181genes	7.63E-20

Supplemental Table S5.

DE genes and probe level data from microarray analysis in adult male zebrafish exposed to 5.8 nM DEHP relative to controls that overlap with genes up-regulated by activation of the mTORC1 pathway. Human Entrez gene IDs were mapped to zebrafish via Ensembl protein homology. Co-expression analysis was then performed using ToppFun. Genes up-regulated through activation of mTORC1 complex were identified using the MSigDB H: Hallmark Gene Sets (v6.0)

(Table available as an excel file)

Supplemental Table S6. DE genes from microarray analysis in adult male zebrafish exposed to 5.8 nM DEHP relative to controls associated with enriched biological pathways (Metabolic Pathways; Fatty acid, triacylglycerol, and ketone body metabolism; FOXA2 and FOXA3 transcription factor networks; Metabolism of amino acids and derivatives; Metabolism of Lipids and Lipoproteins). Genes with the greatest fold change difference in DEHP exposed relative to control are presented. The zebrafish Entrez Gene ID, Agilent array Probe ID, gene symbol, gene name, the human homolog, log2 ratio and fold change are presented.

(Table available as an excel file)

Supplemental Table S7: The Top 50 GO: Biological Process enrichments identified through ToppFun for DE genes differentially expressed in the livers of zebrafish exposed to EE2 (FDR <0.4) as determined by RNA-Seq.

GO Term	q-value Bonferroni
small molecule biosynthetic process	2.27E-10
lipid metabolic process	7.03E-10
single-organism biosynthetic process	1.27E-09
lipid biosynthetic process	3.87E-09
monocarboxylic acid metabolic process	9.71E-09
cellular lipid metabolic process	4.95E-08
fatty acid metabolic process	7.42E-08
triglyceride metabolic process	8.60E-08
cofactor metabolic process	2.31E-07
acylglycerol metabolic process	2.71E-07
neutral lipid metabolic process	2.93E-07
carboxylic acid metabolic process	8.31E-07
organic acid metabolic process	1.14E-06
oxoacid metabolic process	5.48E-06
cholesterol metabolic process	9.28E-06
organic acid biosynthetic process	1.24E-05
carboxylic acid biosynthetic process	1.24E-05
secondary alcohol metabolic process	1.28E-05
fatty acid biosynthetic process	1.85E-05
sterol metabolic process	2.09E-05

long-chain fatty-acyl-CoA biosynthetic process	3.47E-05
monocarboxylic acid biosynthetic process	4.09E-05
fatty-acyl-CoA biosynthetic process	4.68E-05
long-chain fatty-acyl-CoA metabolic process	6.21E-05
cholesterol biosynthetic process	1.35E-04
secondary alcohol biosynthetic process	1.35E-04
steroid metabolic process	1.42E-04
fatty-acyl-CoA metabolic process	1.52E-04
sterol biosynthetic process	2.39E-04
coenzyme metabolic process	2.64E-04
thioester biosynthetic process	4.44E-04
acyl-CoA biosynthetic process	4.44E-04
regulation of lipid biosynthetic process	6.01E-04
cofactor biosynthetic process	1.69E-03
lipid homeostasis	2.08E-03
triglyceride biosynthetic process	2.71E-03
cellular lipid catabolic process	3.33E-03
neutral lipid biosynthetic process	3.74E-03
acylglycerol biosynthetic process	3.74E-03
oxidation-reduction process	5.10E-03
thioester metabolic process	7.17E-03
acyl-CoA metabolic process	7.17E-03
long-chain fatty acid transport	8.73E-03
alcohol biosynthetic process	9.26E-03

response to nutrient levels	1.12E-02
glycerolipid metabolic process	1.43E-02
organic hydroxy compound biosynthetic process	1.47E-02
alcohol metabolic process	1.49E-02
steroid biosynthetic process	1.49E-02
tetrapyrrole metabolic process	1.79E-02

Supplemental Table S8. The Top 50 GO: Biological Process enrichments identified through ToppFun for DE genes differentially expressed in the livers of zebrafish exposed to DEHP (FDR <0.4) as determined by RNA-Seq.

GO Term	q-value Bonferroni
cotranslational protein targeting to membrane	1.01E-19
SRP-dependent cotranslational protein targeting to membrane	1.33E-17
protein localization to endoplasmic reticulum	2.23E-17
protein targeting to ER	1.03E-16
nuclear-transcribed mRNA catabolic process, nonsense-mediated decay	1.79E-16
establishment of protein localization to endoplasmic reticulum	2.41E-16
small molecule biosynthetic process	4.33E-16
organonitrogen compound biosynthetic process	7.77E-16
translational initiation	2.84E-15
peptide biosynthetic process	6.43E-14
translation	9.04E-14
nuclear-transcribed mRNA catabolic process	9.88E-14
amide biosynthetic process	1.07E-13
rRNA processing	3.24E-13
organic acid metabolic process	3.79E-13
peptide metabolic process	4.09E-13
mRNA catabolic process	5.81E-13
protein targeting to membrane	6.18E-13
rRNA metabolic process	6.23E-13

ribosome biogenesis	7.72E-13
cellular amide metabolic process	7.84E-13
carboxylic acid metabolic process	1.81E-12
single-organism biosynthetic process	4.15E-12
viral transcription	9.55E-12
RNA catabolic process	1.01E-11
oxoacid metabolic process	2.09E-11
viral gene expression	3.28E-11
organic substance catabolic process	1.28E-10
multi-organism metabolic process	2.53E-10
establishment of protein localization to membrane	3.42E-10
monocarboxylic acid metabolic process	3.53E-10
cellular nitrogen compound catabolic process	1.05E-09
heterocycle catabolic process	1.17E-09
ribonucleoprotein complex biogenesis	1.45E-09
cholesterol metabolic process	1.90E-09
aromatic compound catabolic process	1.99E-09
organic cyclic compound catabolic process	2.10E-09
cofactor metabolic process	2.67E-09
cellular catabolic process	2.74E-09
secondary alcohol metabolic process	3.47E-09
nucleobase-containing compound catabolic process	3.75E-09
ncRNA processing	8.32E-09
sterol metabolic process	8.69E-09

lipid metabolic process	1.35E-08
lipid biosynthetic process	1.62E-08
carboxylic acid biosynthetic process	1.17E-07
organic acid biosynthetic process	1.17E-07
fatty acid metabolic process	1.18E-07
ncRNA metabolic process	1.34E-07
cofactor biosynthetic process	2.25E-07

Supplemental Table S9. The Top 50 co-expressed gene signatures identified through ToppFun for DE genes differentially expressed in the livers of zebrafish exposed to EE2 (FDR <0.4) as determined by RNA-Seq.

Co-Expression	q-value Bonferroni
Transcripts dependent upon IRS1 and IRS2 [GeneID=3667, 8660] for normal expression in liver.	3.47E-07
Genes up-regulated through activation of mTORC1 complex.	6.34E-07
Genes which best discriminate between two groups of breast cancer according the status of ESR1 and AR [GeneID=2099;367]: apocrine (ESR1- AR+) vs basal (ESR1- AR-).	7.44E-07
Genes up-regulated in liver from mice transgenic for SREBF1 or SREBF2 [GeneID = 6720, 6721] and down-regulated in mice lacking SCAP [GeneID=22937].	1.39E-06
Genes up-regulated in E12.5 forelimb buds with POR [GeneID=5447] knockout.	1.80E-06
Genes changed in NIH 3T3 cells (embryonic fibroblast) by expression of one or more of C/EBP proteins: CEBPA, CEBPB, CEBPG, and CEBPD [GeneID=1050;1051;1054;1052].	3.35E-06
Liver selective genes	5.25E-06
Genes encoding proteins involved in processing of drugs and other xenobiotics.	1.17E-05
Genes up-regulated in primary cultures of ovarian surface epithlium cells exposed to progesterone [PubChem=5994] for 5 days.	1.77E-05
Genes down-regulated in hepatocellular carcinoma (HCC) compared to normal liver samples.	3.12E-05
Genes involved in cholesterol homeostasis.	4.06E-05
Genes down-regulated in APL (acute promyeolocytic leukemia) blasts expressing PML-RARA fusion [GeneID=5371;5914] compared to normal promyeloblasts.	5.13E-05
Genes encoding proteins involved in metabolism of fatty acids.	4.65E-04

Genes up-regulated in Daudi cells (B lymphocytes) stably expressing CD5 [GeneID=921] off a plasmid vector.	5.33E-04
Adipocyte abundant genes down-regulated in 3T3-L1 cells (fibroblasts induced to differentiate to adipocytes) in response to TNF [GeneID=7124].	2.70E-03
Genes up-regulated in comparison of dendritic cells (DC) versus effector memory CD4 [GeneID=920] T cells.	2.82E-03
'Early-TGFB1 signature': genes overexpressed in primary hepatocytes at an early phase of TGFB1 [GeneID=7040] treatment; is associated with a less invasive phenotype.	3.05E-03
Selected genes up-regulated during differentiation of 3T3-L1 cells (fibroblast) into adipocytes in response to adipogenic hormones.	4.19E-03
Genes up-regulated in circulating endothelial cells (CEC) from cancer patients compared to those from healthy donors.	7.57E-03
Genes which best discriminate between two groups of breast cancer according to the status of ESR1 and AR [GeneID=2099;367]: apocrine (ESR1- AR+) and luminal (ESR1+ AR+).	1.18E-02
Molecular timetable composed of 162 time-indicating genes (182 probes) in the peripheral (liver) clock.	1.19E-02
Genes up-regulated upon overexpression of PARVB [GeneID=29780] in MDA-MB-231 cells (breast cancer) cultured in 3D Matrigel only.	1.43E-02
Genes down-regulated in hepatocellular carcinoma (HCC) from MYC and E2F1 [GeneID=4609;1869] double transgenic mice.	1.61E-02
Genes from 'subtype S3' signature of hepatocellular carcinoma (HCC): hepatocyte differentiation.	2.37E-02
Up-regulated at 48-96 h during differentiation of 3T3-L1 cells (fibroblast) into adipocytes.	2.91E-02
Genes up-regulated in MCV152 cells (ovarian cancer) treated with follicle stimulating hormone (FSH).	3.30E-02
Human orthologs of genes down-regulated in zebra fish after knockdown of BMYB [GeneID=4605] by morpholino.	3.48E-02

Genes up-regulated in comparison of macrophages versus NK cells.	3.60E-02
Genes down-regulated in comparison of dendritic cells (DC) exposed to 50 worms/well B. malayi versus DC exposed to M. tuberculosis	3.60E-02
Genes down-regulated in control macrophages: untreated versus primed by IFNG [GeneID=3458].	3.60E-02
Genes down-regulated in comparison of dendritic cells (DC) exposed to L. donovani versus DCs exposed to M. tuberculosis.	3.60E-02
Genes negatively correlated with recurrence free survival in patients with hepatitis B-related (HBV) hepatocellular carcinoma (HCC).	4.84E-02
IRF4 [GeneID=3662] target genes induced after activation of primary B lymphocytes by anti-IgM crosslinking.	5.15E-02
Cluster 1: genes whose up-regulation peaked one day after knockdown of OPN [GeneID=6696] by RNAi in the NIH3T3 cells (fibroblasts) transformed by activated HRAS [GeneID=3265].	5.96E-02
Genes with promoters bound by both PPARG and RXRA [GeneID=5468, 6256] at 8 day time point of adipocyte differentiation of 3T3-L1 cells (preadipocyte).	6.51E-02
Genes up-regulated in liver from mice with liver specific knockout of POR [GeneID=5447].	6.72E-02
Genes whose expression changes in Calu-6 cells (lung cancer) by TNF [GeneID=7124] were blocked completely by p38 inhibitor LY479754.	8.19E-02
Genes down-regulated in adipose tissue from obese mouse strains compared to the lean ones.	1.27E-01
Adipocyte genes induced in 3T3-L1 cells (adipocyte) by constitutively active PPARG [GeneID=5468] or its agonist, TZD [PubChem=5437].	1.40E-01
Genes down-regulated in late serum response of CRL 2091 cells (foreskin fibroblasts).	1.55E-01
Genes up-regulated by everolimus [PubChem = 6442177] in prostate tissue.	1.60E-01
Genes up-regulated in ME-A cells (breast cancer) undergoing apoptosis in response to doxorubicin [PubChem=31703].	2.24E-01
Genes down-regulated in hepatoblastoma samples compared to normal liver tissue.	2.31E-01

Genes up-regulated in A549 cells (lung adenocarcinoma) upon SKIL [GeneID=6498] knockdown by RNAi.	2.33E-01
Genes highly expressed in hepatocellular carcinoma with good survival.	2.57E-01
Genes up-regulated in MCF-7 cells (breast cancer) positive for ESR1 [Gene ID=2099] and engineered to express ligand-activatable EGFR [Gene ID=1956].	3.24E-01
Genes down-regulated during pubertal mammary gland development between week 4 and 5.	3.53E-01
Genes up-regulated in CEM-C1 cells (T-CLL) by everolimus [PubChem = 6442177], an mTOR pathway inhibitor.	3.53E-01
Human orthologs of genes up-regulated in the crb ('crash and burn') zebrafish mutant that represents a loss-of-function mutation in BMYB [GeneID=4605].	3.63E-01
Genes down-regulated in comparison of CD4 [GeneID=920] T cells treated with IL4 [GeneID=3565] and anti-IL12 at 0.5 h versus those at 72 h.	3.74E-01

Supplemental Table S10. The Top 50 co-expressed gene signatures identified through ToppFun for DE genes differentially expressed in the livers of zebrafish exposed to DEHP (FDR <0.4) as determined by RNA-Seq.

Co-Expression	q-value Bonferroni
Genes up-regulated in liver from mice transgenic for SREBF1 or SREBF2 [GeneID = 6720, 6721] and down-regulated in mice lacking SCAP [GeneID=22937].	3.09E-16
Molecular timetable composed of 162 time-indicating genes (182 probes) in the peripheral (liver) clock.	2.41E-14
Genes up-regulated through activation of mTORC1 complex.	6.79E-14
Genes up-regulated in E12.5 forelimb buds with POR [GeneID=5447] knockout.	4.72E-12
Genes involved in cholesterol homeostasis.	6.58E-12
Genes up-regulated in polymorphonuclear leukocytes (24h): control versus infection by A. phagocytophilum.	1.22E-11
Genes with increased copy number that correlates with increased expression across six different lung adenocarcinoma cell lines.	1.57E-11
Housekeeping genes identified as expressed across 19 normal tissues.	3.36E-11
Genes from 'subtype S3' signature of hepatocellular carcinoma (HCC): hepatocyte differentiation.	3.73E-11
Transcripts dependent upon IRS1 and IRS2 [GeneID=3667, 8660] for normal expression in liver.	5.31E-10
Liver selective genes	6.57E-10
Genes down-regulated in dendritic cells: untreated versus 2h after infection of Leishmania major.	1.75E-09
Genes up-regulated in primary cultures of ovarian surface epithlium cells exposed to progesterone [PubChem=5994] for 5 days.	2.27E-09
Down-regulated genes in the canonical gene expression signature of the fibroblast core serum response (CSR) defined by the Stanford group.	3.88E-09

Genes down-regulated in polymorphonuclear leukocytes (9h): control versus infection by A. phagocytophilum.	1.92E-08
Genes down-regulated in dendritic cells: untreated versus 4h after infection of Leishmania major.	1.92E-08
Genes up-regulated in liver from transgenic mice with reduced expression of POR [GeneID=5447] in all tissues.	8.33E-08
Genes up-regulated in Daudi cells (B lymphocytes) stably expressing CD5 [GeneID=921] off a plasmid vector.	1.50E-07
Genes up-regulated in RPTEC cells (normal kidney) by hypoxia.	5.14E-07
Genes encoding proteins involved in metabolism of fatty acids.	6.22E-07
Genes up-regulated upon overexpression of PARVB [GeneID=29780] in MDA-MB-231 cells (breast cancer) cultured in 3D Matrigel only.	7.54E-07
Genes up-regulated in NHEK cells (normal epidermal keratinocytes) after UVB irradiation.	1.67E-06
Genes down-regulated in KLRG1 low [GeneID=10219] CD8 T effector cells during infection: ID2 [GeneID=3398] knockout versus ID2 and BCL2L11 [GeneID=3398;10018] knockout.	1.89E-06
Genes up-regulated in the Kras2LA mouse lung cancer model with mutated KRAS [GeneID=3845].	1.91E-06
Genes up-regulated in liver from mice with liver specific knockout of POR [GeneID=5447].	1.91E-06
Genes down-regulated in hepatocellular carcinoma (HCC) compared to normal liver samples.	2.46E-06
Genes changed in NIH 3T3 cells (embryonic fibroblast) by expression of one or more of C/EBP proteins: CEBPA, CEBPB, CEBPG, and CEBPD [GeneID=1050;1051;1054;1052].	4.08E-06
Genes whose promoters are bound by MYC [GeneID=4609], according to MYC Target Gene Database.	4.54E-06
Adipocyte abundant genes down-regulated in 3T3-L1 cells (fibroblasts induced to differentiate to adipocytes) in response to TNF [GeneID=7124].	6.75E-06
Genes down-regulated in uveal melanoma: class 2 vs class 1 tumors.	7.30E-06

Genes down-regulated in P14 nerves of transgenic mice having hypomorhic (reduced function) allele of EGR2 [GeneID=1959].	8.02E-06
'Early-TGFB1 signature': genes overexpressed in primary hepatocytes at an early phase of TGFB1 [GeneID=7040] treatment; is associated with a less invasive phenotype.	1.27E-05
Genes that contributed maximally to the GSEA score of the up-regulated gene set from the KrasLA mouse model in two human lung cancer expression data sets comparing mutant vs normal KRAS [GeneID=3845].	1.27E-05
Genes up-regulated in follicular helper T cells: BCL6 [GeneID=604] high versus BCL6 [GeneID=604] low.	1.69E-05
Genes up-regulated in CD11b+ cells from spleen of healthy C57BL6 mice versus CD11b+ cells from tumor infiltrating monocytes of BALB/c mice bearing 4T1 mammary carcinoma.	1.69E-05
Genes up-regulated in comparison of naive CD4 [GeneID=920] CD8 T cells versus unstimulated dendritic cells (DC).	1.69E-05
Genes up-regulated in comparison of peripheral blood mononuclear cells (PBMC) from healthy donors versus PBMC from patients with acute influenza infection.	2.03E-05
Selected genes up-regulated in response to the Ras inhibitor salirasib [PubChem=5469318] in a panel of cancer cell lines with constantly active HRAS [GeneID=3265].	3.64E-05
Genes down-regulated in peripheral blood mononuclear cells (PBMC) from sickle cell disease patients compared to those from healthy subjects.	3.66E-05
Hepatic graft versus host disease (GVHD), day 7: down-regulated in allogeneic vs syngeneic bone marrow transplant.	4.26E-05
Down-regulated genes in MDA-MB-435 cells (breast cancer) undergoing G2/M arrest after treatment with 2-methoxyestradiol (2ME2) [PubChem=1573].	5.21E-05
Genes which best discriminate between two groups of breast cancer according to the status of ESR1 and AR [GeneID=2099;367]: apocrine (ESR1- AR+) and luminal (ESR1+ AR+).	5.55E-05
Genes down-regulated in bone marrow-derived dendritic cellstreated by poly(IC): 1h versus 24h.	7.72E-05
Up-regulated genes in the subpopulation of invasive PyMT cells (breast cancer)	8.14E-05

compared to the general population of PyMT cells.	
Genes up-regulated in Vd2 gamma delta T cells: untreated versus LPS.	1.04E-04
Human orthologs of genes up-regulated in the crb ('crash and burn') zebrafish mutant that represents a loss-of-function mutation in BMYB [GeneID=4605].	1.16E-04
Genes up-regulated in macrophages: classical (M1) versus alternative (M2).	1.24E-04
Genes encoding proteins involved in processing of drugs and other xenobiotics.	1.41E-04
Genes up-regulated in thymocytes: double positive versus CD4 [GeneID=920] single positive.	1.41E-04
Genes down-regulated in double positive thymocytes: wildtype versus TCF3 and TCF12 [GeneID=6929;6938] knockout.	1.41E-04

Supplemental Table S11. DE genes from RNAseq analysis in adult male zebrafish exposed to 5.8 nM DEHP relative to controls associated with enriched biological pathways (Metabolic Pathways; Fatty acid, triacylglycerol, and ketone body metabolism; FOXA2 and FOXA3 transcription factor networks; Metabolism of amino acids and derivatives; Metabolism of Lipids and Lipoproteins) are presented. The Ensembl_gene_ids for zebrafish, and the corresponding human homolog (from Ensembl) are listed. The baseMean, log2FoldChange, lfcSE, stat, pvalue and padj as determined by DESeq2 are listed.

(excel table provided)
